# Development and pilot evaluation of a large language model-based clinical simulation system for medical education in gastroenterology

**DOI:** 10.1186/s12909-026-09709-3

**Published:** 2026-06-25

**Authors:** Stiliano Maimaris, Gianmarco Tedino, Giusi Aurora Memoli, Carlotta Crisciotti, Alessandra Albrizio, Camilla Sammartino, Erica Bartolotta, Giulio Massetti, Giulia Mantica, Paolo Minerba, Federico Biagi, Annalisa Schiepatti

**Affiliations:** 1 Department of Internal Medicine and Therapeutics, University of Pavia, Pavia, Italy; 2 Istituti Clinici Scientifici Maugeri IRCCS, Gastroenterology Unit of Pavia Institute, Via Salvatore Maugeri 10, Pavia, 27100 Italy; 3 Digestive Endoscopy Unit, ASST Pavia-Ospedali Voghera-Vigevano, Pavia, Italy

**Keywords:** Large language models, Artificial intelligence, Medical education, Clinical simulation, Clinical reasoning, Virtual patient

## Abstract

**Background:**

Medical education relies on experience to develop clinical reasoning skills, yet patient access is often limited. Large language models (LLMs) offer an accessible alternative for simulating patient encounters. We aimed to develop and evaluate an LLM-based clinical simulation system for gastroenterology that simulates entire clinical encounters in natural language, from history-taking through to treatment and follow-up planning.

**Methods:**

The study was conducted in three phases. First, a system comprising an LLM-based clinical case generator, an LLM-based patient simulator, and a web chat interface was developed and iteratively refined. Second, two gastroenterology fellows performed 20 clinical case simulations each as initial testers, providing feedback that guided further system adjustments. Third, six independent gastroenterology fellows, blinded to the development process, evaluated the final system by completing the same 20 clinical simulations. All evaluators rated the system using a 5-point Likert scale across four domains: case presentation accuracy, adaptability to user interactions, realism, and educational value.

**Results:**

A total of 160 evaluations were collected across 20 cases and 8 raters. At final evaluation, all domains scored highly: educational value (mean 4.57 ± 0.63), accuracy (4.42 ± 0.63), adaptability (4.33 ± 0.75), and realism (4.32 ± 0.81). Scores differed significantly across domains (*p* < 0.01), with educational value rated highest. Evaluation panellists rated all domains significantly higher than initial testers (all *p* < 0.01) at an exploratory comparison.

**Conclusions:**

The LLM-based clinical simulator demonstrated high accuracy, realism, adaptability, and educational value for gastroenterology case simulation in this pilot evaluation, and may represent an accessible and cost-effective tool for clinical reasoning training.

**Supplementary Information:**

The online version contains supplementary material available at https://doi.org/10.1186/s12909-026-09709-3.

## Background

Medical education requires interaction with patients to acquire clinical reasoning skills, but access to real patients can often be limited [1]. Simulation-based methods can provide additional clinical reasoning practice in a safe, controlled environment, and have historically employed actors trained to portray medical conditions consistently, known as standardized patients [1, 2]. While standardised patients have previously been shown to be cost-effective [1], they also have several limitations including training requirements, cost, and issues with scheduling and limited availability.

Modern Large Language Models (LLMs) are trained on extremely large amounts of diverse data and are able to produce high-quality realistic responses to prompts across very diverse domains. This was first exemplified by GPT-4, a landmark model released by OpenAI which exhibited emergent abilities across many domains including mathematics, coding, vision, law, psychology, but also medicine [3], also achieving sufficient levels of medical knowledge to pass medical licensing examinations such as the United States Medical Licensing Examination (USMLE) [4].

The recent rapid advancements in artificial intelligence, particularly LLMs, have opened new avenues for realistic, scalable, and cost-effective simulation training for medical education. LLM-based chatbots can produce human-like textual responses that can allow learners to interact with a simulated ‘patient’ through typed or spoken conversations. In this regard, a recent randomised controlled trial showed that patient simulation systems based on LLMs can improve the clinical decision-making of medical students [5], confirming findings from previous virtual patient simulation approaches based on traditional natural language processing software [6, 7]. Moreover, although some limitations remain, it has been shown that it is feasible to dynamically create good quality content for virtual patient simulations using LLMs [8].

The theoretical advantages of LLM-based simulations include 24/7 availability, standardized yet adaptable case presentations across diverse clinical scenarios including rare diseases, with no need for real-patient data, and cost-effectiveness at scale. Several recent studies have shown the feasibility of using LLMs as standardised patients for simulation purposes with positive results [9–11]. Although recent studies have mainly been focused on the feasibility of the approach, LLMs have been shown to exhibit excellent levels of accuracy from a medical point of view [12].

In light of these recent technological advances, we aimed to develop an LLM-based system to generate realistic and detailed gastroenterology clinical cases and present them as a virtual patient to simulate clinician-patient interactions completely in natural language, starting from history-taking, to diagnostic reasoning, test selection and interpretation, to treatment and follow-up planning. We then aimed to evaluate the clinical accuracy, realism, adaptability to diverse prompting, and educational value of the developed system.

## Methods

### Study design

The study was conducted in three phases: In the first phase the clinical simulator was developed and iteratively refined. Then in a second phase, initial testing and evaluation of the system was performed by two testers (GAM, CC). Based on feedback from the initial testers, adjustments were made to the system to improve the quality of the simulation system. Finally, in the third phase a final pilot evaluation panel composed of six gastroenterology fellows (AA, CS, EB, GM, GM, PM), who were blinded to and not involved in the development and initial evaluation phases, independently evaluated the final version of the system.

### Simulator development phase

**Fig. 1:**
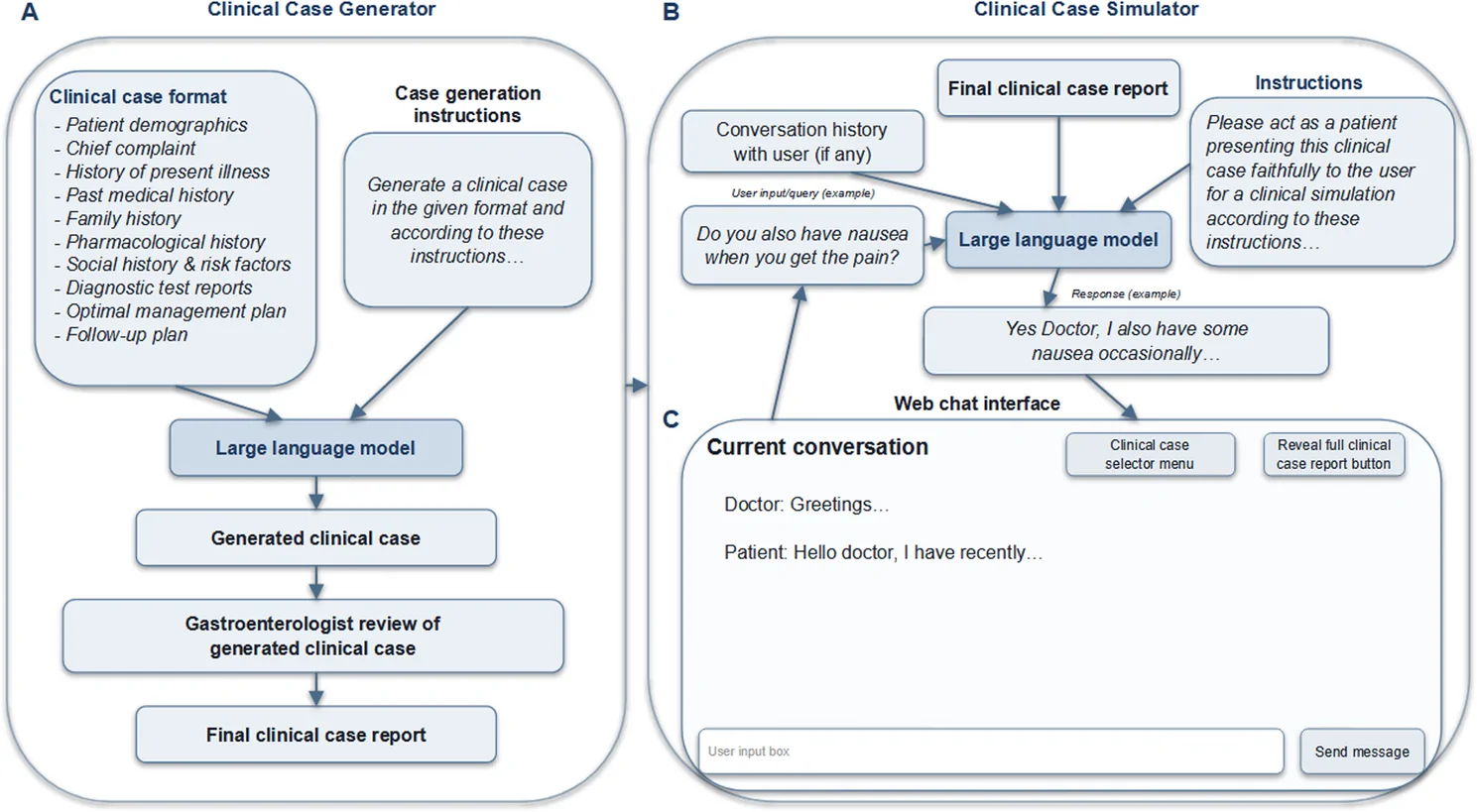
Overview of the architecture of the clinical simulator system. Panel **A**) Clinical case generator system that generates the clinical case report for use by the simulator; panel **B**) mechanism underlying the functioning of the clinical simulator system; panel **C**) web interface for interacting with the clinical simulator using text or voice

An LLM-based clinical simulator was developed to generate and present clinical cases via a web-based interface. Figure 1 illustrates components of the clinical simulator and provides an overview of its functioning. Overall, the simulator consists of three main components: 1) an LLM-based clinical case generator; 2) an LLM-based patient simulation system that presents each underlying clinical case interactively to the user; 3) a web chat-based interface allowing the user to interact with the system.

#### Clinical case generator

The LLM-based clinical case generator (see Fig. 1) was developed to generate diverse clinical cases in a standardised format containing the following key information: patient demographics, chief complaint and history of present illness, past medical history, family history, pharmacological history, social history and risk factors, physical examination findings, diagnostic test reports (including laboratory tests, endoscopic examinations, histopathology reports, radiology reports, and any other tests relevant to the clinical case), the final diagnosis, and an optimal management and follow-up plan for case review after completion. A standardised template was provided to the LLM as part of the generation prompt to guide the response format of the generated cases. Exact prompts, the standardised template and generation settings (e.g. temperature) are included in supplementary materials.

The clinical case generation system is based on providing an LLM with a specific response format for clinical case generation together with instructions for clinical case generation. The generation system was built to be adaptable and able to generate cases either completely randomised or based on more specific instructions (e.g. based on a brief description of the case to generate, while filling in the missing details in a way consistent with the description). For generation from a brief description of the case the LLM is prompted directly to generate a clinical case from the description in the format of the standardised template. Instead, for fully randomised case generation the generation pipeline consists of first prompting the LLM to generate 100 one-sentence brief descriptions of the case in an XML structure reply format, then using a random number generator to randomly select one of the 100 brief descriptions. This randomly selected brief case description is then passed as input to the full clinical case generation system. Further technical details on the generation pipeline are provided in supplementary materials. During the development phase, several LLMs available at the time were evaluated for the clinical case generator role, including both open-weights models (Llama 3.3 70B, Gemma 3 27B, DeepSeek R1) and proprietary models (GPT-4o, o4-mini, Claude Sonnet 3.7, Gemini 2.5 Pro, Gemini 2.0 Flash), before selecting Gemini 2.5 Pro (model version gemini-2.5-pro-preview-03-25 at time of the study) based on high output quality, clinical accuracy, excellent instruction following and low hallucination rate for use in the testing and evaluation phases of the study. We specify that for the purposes of the study all the generated clinical cases were also then reviewed by an experienced gastroenterologist to check for any medical errors in the generated case reports. Evaluators were also asked to report any errors they detected in the clinical cases during the case review phase.

#### Patient simulation system

As shown in Fig. 1, we then developed an LLM-based system that, given instructions to act as a virtual patient based on a provided underlying clinical case report, would be able to interact appropriately with the user, simulating a realistic patient-clinician interaction. During the development phase, several LLMs and sets of system instructions were iteratively tested for the patient simulation role. The prompt instructs the model to act as a virtual patient based on a provided clinical case report, respond naturally to clinician questions, provide information only when appropriately elicited, and simulate realistic patient behaviour including appropriate emotional responses. Gemini-2.0-flash was selected for the patient simulator based on its good instruction-following, fast response latency (~ 0.30 s), and low cost. The complete prompts and generation parameters (e.g. temperature) are provided in supplementary materials.

#### Web chat interface

**Fig. 2:**
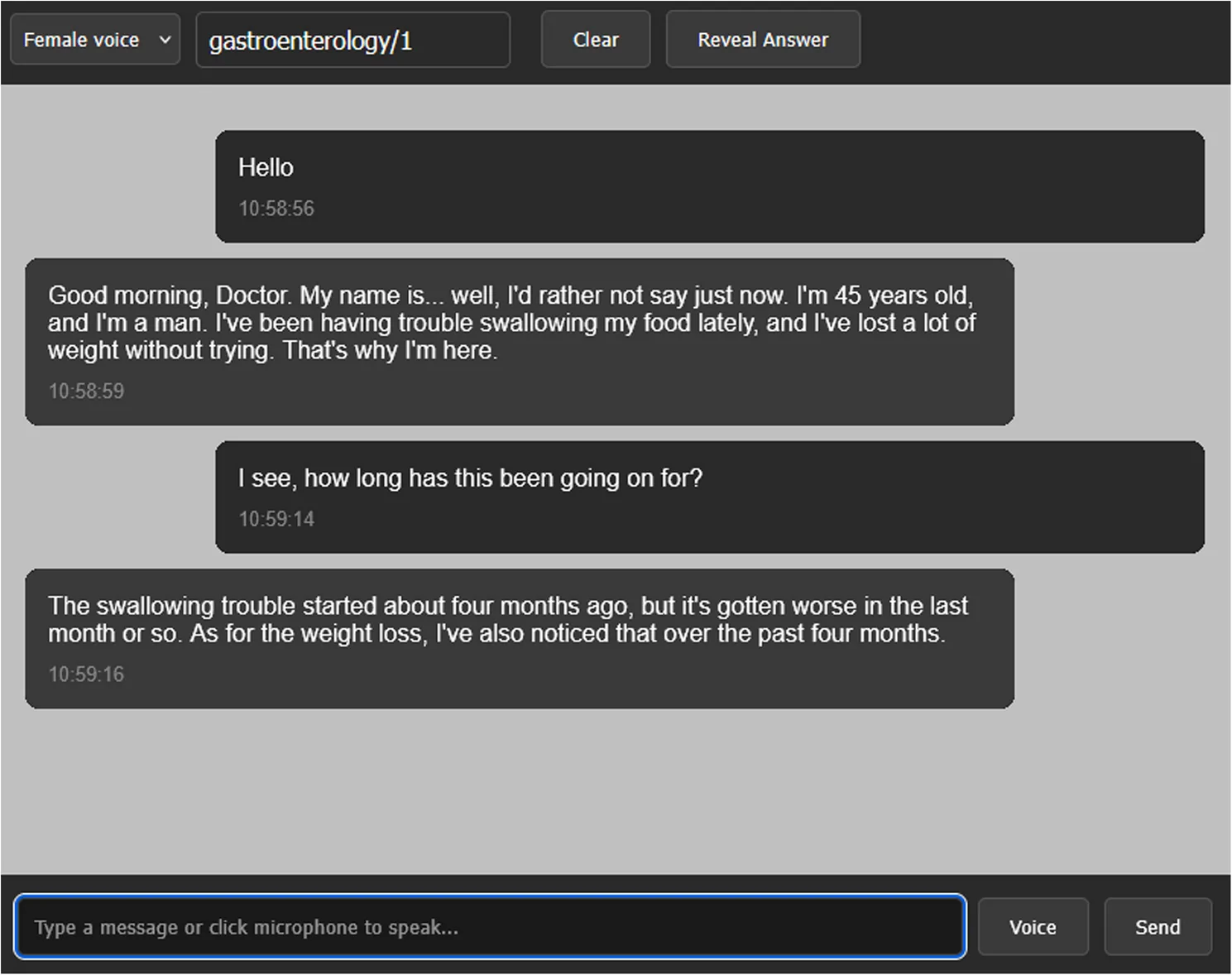
Snapshot of the web interface for interacting with the clinical simulator via text or voice

A simple easy-to-use web interface was developed to allow users to interact with the underlying system (a snapshot of the interface is shown in Fig. 2). The web interface was designed to allow user interaction either via text input or voice input, with the latter utilising the whisper-large-v3 model [13] for automatic transcription of user speech to text. The whisper model is multilingual and allows for fast real-time automatic conversion of voice input to text, allowing users to interact with the simulator also using voice. Additionally, the interface provides a menu to either select from pre-generated clinical cases by entering the case number or the option to generate a new random clinical case. It also includes a button to reveal the underlying clinical case report for review after the clinical case simulation has been completed.

### Initial testing and preliminary evaluation phase

During the initial testing phase, two gastroenterology fellows (GAM, CC) each performed 20 clinical case simulations using the system and provided feedback on issues with the system and possible improvements. Based on the feedback, adjustments were then made to the system. In addition to providing feedback to improve the system, the initial testers also performed a preliminary evaluation of the system using a standardised evaluation form developed specifically for this study. The form comprised 5-point Likert-scale items (1 = poor, 5 = excellent) across four domains: presentation accuracy, adaptability to user interactions, realism of the simulation, and perceived educational value. Two additional open-ended free-text fields solicited feedback on errors detected, suggestions, and general qualitative comments. Responses were recorded via a Google Form. The evaluation tool was first pilot-tested (by SM and GT) during development, although the tool did not undergo a formal calibration process.

### Final pilot evaluation phase

After the development and initial testing phases were completed a final pilot evaluation panel consisting of 6 gastroenterology fellows (AA, CS, EB, GM, GM, PM) from the gastroenterology fellowship programme of the University of Pavia (1st- to 3rd-year fellows at the time of the study), who were not involved in the development and initial testing phases, performed the same 20 clinical simulations used in the initial testing phase to re-evaluate the final adjusted version of the system.

After completing each simulation evaluation panellists were asked to also read the underlying clinical case report to assess it for errors or inconsistencies with the simulator’s responses. They were then asked to evaluate the system using the same standardised evaluation form used for the initial testing phase, comprising 5-point Likert-scale ratings (1 = poor, 5 = excellent) across the four domains and open-ended free-text fields for qualitative feedback on errors, suggestions for improvement, and general qualitative comments.

### Statistical analysis

Statistical analysis was conducted using R version 4.3.1 (R Foundation for Statistical Computing, Vienna, Austria). Descriptive statistics were used to summarize the 5-point Likert scale ratings for each of the four evaluated domains: presentation accuracy, adaptability to user interactions, realism of the simulation, and educational value. For each domain, mean±standard deviation (SD) were calculated across all simulations. Frequency distributions for each point on the Likert scale (1 to 5) were also calculated for each domain to provide a comprehensive overview of the evaluators’ ratings. Ratings across domains were compared using the Friedman test and post-hoc comparisons with Holm correction for multiplicity. In an exploratory analysis to compare the clinical simulator’s performance between the initial testing phase and the final pilot evaluation phase while accounting for the clustering of data among raters, evaluation scores were aggregated at the clinical case level. Specifically, the median score assigned to each of the 20 distinct clinical cases was calculated separately for the initial testers and the final pilot evaluation panellists. The distributions of these 20 matched pairs of median scores were then compared for each domain using the Wilcoxon signed-rank test. For all analyses *p*-values of < 0.05 were considered statistically significant.

## Results

### System development, preliminary testing, model selection and clinical case generation

Figure 1 shows an overview of the architecture of the system. During the development phase, preliminary testing of the system was performed to adjust the system, identify and correct any technical issues, and select the final models to use for the clinical case generator and clinical case simulator parts of the system for the purposes of this study. During the development phase (April 2025), we experimented with several LLMs available at the time, including both major open-weights models (e.g., Llama 3.3 70B, Gemma 3 27B, DeepSeek R1) and closed proprietary models (e.g., GPT-4o, o4-mini, Claude Sonnet 3.7, Grok 3 mini, Gemini 2.5 and 2.0 models).

Overall, based on our testing we observed that ‘reasoning’ models (e.g. Gemini-2.5-pro and o4-mini) performed best for the clinical generator part of our system due to high quality responses with a very low rate of errors and hallucinations. However, they were not suitable for use for the clinical simulator part of the system mainly due to long response latency (> 30s) due to the ‘thinking time’ that these models require before responding. Instead, for the clinical simulator part of the system we found smaller inexpensive non-reasoning models (e.g. Gemini-2.0-flash, GPT-4o, Llama 3.3 70B) performed best mainly due to fast response times allowing real-time interactive use. We also noted significantly lower costs with these models compared to reasoning models which may be relevant when deploying the system to a large user base or on local hardware without data-centre level hardware.

Based on technical specifications and our preliminary testing results, Gemini-2.5-pro was selected for use in the clinical case generator due to high benchmark scores on reasoning and knowledge-based benchmarks and low hallucination rate [14], while Gemini-2.0-flash was selected for use in the clinical simulator part of the system due to a combination of good scores on instruction-following benchmarks, very fast response time (~ 0.30s), and extremely low cost ($0.60 per 1 million output tokens) [15], allowing real-time interactive and cost-effective use of the simulator system.

As part of system development and preliminary testing, clinical cases were generated using the clinical case generator system and were then all reviewed by an experienced gastroenterologist to identify areas for improvement and any medical errors in the generated cases. The clinical case template and case generator system were iteratively refined until results appeared satisfactory. Technical issues were also identified and resolved. Once the clinical case generator, the simulator and web interface all appeared to be functioning correctly, 20 complete clinical cases were generated in April 2025 using the finalised case generator system. These cases were generated once, reviewed and fixed, and then used identically in both the initial testing and final evaluation phases. These 20 clinical cases covered diverse areas of gastroenterology including intestinal disorders (6 cases), gastrointestinal malignancy (4 cases), liver disorders (3 cases), pancreatic/biliary disorders (3 cases), oesophageal and gastric disorders (2 cases), and gastrointestinal bleeding (2 cases). These 20 cases were all each assessed for any medical errors or inaccuracies by a gastroenterologist, and none were identified.

### Initial testing phase results

**Fig. 3:**
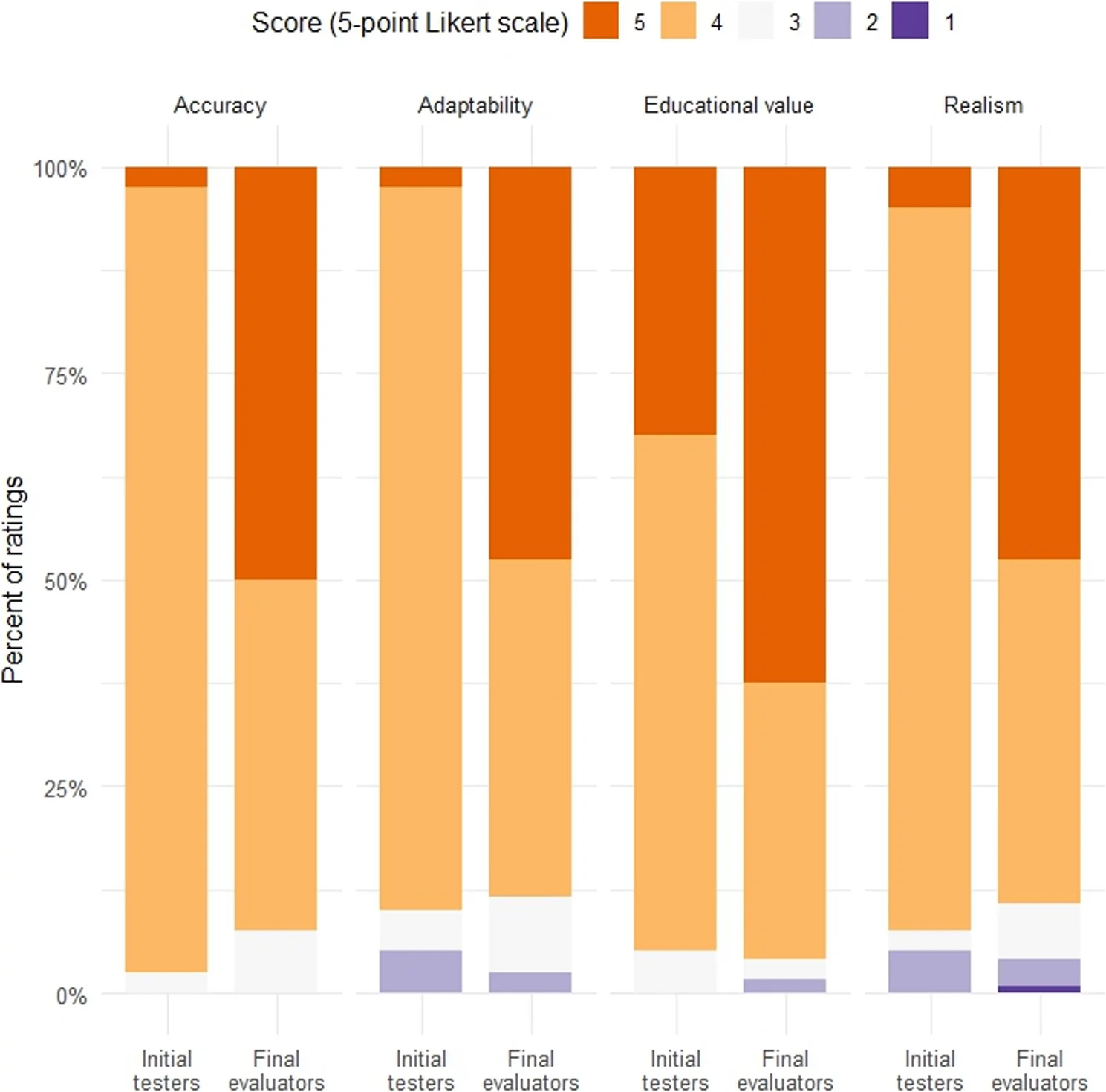
Comparison of the evaluation results given by the initial testers and final evaluation panel for the clinical simulator system

Two initial testers (GAM, CC) completed a total of 40 clinical case simulations and provided detailed feedback on any remaining issues with the clinical simulator not identified during development and preliminary testing. Overall, issues with the system were identified in 9/40 simulations during the initial testing phase. The identified issues were generally minor and included a missing requested test result in 2/40 simulations, the provision of additional test results beyond those requested in 3/40 simulations, inappropriate responses by the simulator in 3/40 simulations, and finally in 1/40 simulations the physical examination results were reported by the simulator informally in the first person rather than described in medical terms in a report format. Despite these minor issues, as shown in Table 1 and Fig. 3, overall the initial testers rated the simulator’s performance favourably across all four domains, with scores of 4 or more in > 80% of cases for all four domains. Based on the identified issues, further minor adjustments were made to the clinical simulator before proceeding further to the final evaluation phase.

**Table 1:** Evaluation of the clinical simulator system during the initial testing phase and by final evaluation panellists

Domain	Initial testers (*N* = 2)	Evaluation panellists (*N* = 6)	*P*-value
Accuracy (mean ± SD)	4.00 ± 0.23	4.42 ± 0.63	< 0.01
Adaptability (mean ± SD)	3.88 ± 0.52	4.33 ± 0.75	< 0.01
Educational value (mean ± SD)	4.28 ± 0.55	4.57 ± 0.63	< 0.01
Realism (mean ± SD)	3.92 ± 0.53	4.32 ± 0.81	< 0.01

*N *number, *SD *standard deviation

### Final pilot evaluation

Finally, the 6 final pilot evaluation panellists completed the same 20 clinical cases used for initial testing with the final version of the clinical simulator after the issues identified during initial testing were addressed. As shown in Table 1 and Fig. 3, the simulator at final evaluation was overall rated very favourably across all four domains. In the exploratory comparison of scores between the initial testing and final evaluation phases, all domain-specific scores at final evaluation were higher (all *p* < 0.01) than ratings given by the initial testers. Educational value was the highest rated domain with a mean score of 4.57, although accuracy, adaptability and realism also scored highly with mean scores of 4.42, 4.33, and 4.32, respectively. Ratings differed significantly across the four domains (Friedman test *p* < 0.01). Post-hoc pairwise comparisons with Holm correction for multiplicity showed that educational value was rated significantly higher than accuracy (*p* < 0.01), adaptability (*p* < 0.01) and realism (*p* < 0.01). Accuracy was rated significantly higher than both adaptability (*p* = 0.03) and realism (*p* = 0.03). No significant difference was found instead between adaptability and realism (*p* = 0.89).

No further significant issues with the simulator were identified during this final pilot evaluation, although some suggestions for further improvements to the system were made by evaluation panellists, such as simulating multidisciplinary case discussion for deciding treatment of gastrointestinal malignancy cases and the addition of intra-procedural endoscopic treatment decision-making (e.g. selection of treatment for gastrointestinal bleeding sources identified during endoscopy). For interested readers, we include in supplementary materials some examples of complete simulation transcripts from the final evaluation phase and the corresponding underlying case reports for each simulation.

## Discussion

We developed and evaluated a clinical case simulator that generates and presents gastroenterology clinical cases with high accuracy, adaptability, and realism. Both initial testers and evaluation panellists rated the system highly, with educational value consistently receiving the highest ratings. Our findings align with and extend previous research on LLMs for clinical simulation [5, 9–11], demonstrating a comprehensive system that automates both case generation and dynamic patient simulation, evaluated through a structured multi-phase process.

In an exploratory comparison, evaluation panellists rated the simulator more favourably than initial testers, suggesting an improvement of the simulator due to feedback from the initial testing phase. However, higher scores cannot be attributed definitively to feedback-driven refinements and may also reflect inter-rater variability to some degree. Also, although suggestions for further improvements were made at final evaluation, no significant issues or errors were reported during final evaluation.

A major strength of our system is its ability to conduct entire simulations in natural language without relying on multiple-choice questions or fixed pathways, closely aligning with everyday clinical practice. The ability to generate realistic, medically accurate cases avoids the need for real patient data collection and anonymization or laborious manual case creation requiring substantial gastroenterologist input. It also enables generation of cases for rare conditions difficult to encounter in practice.

A previous study using GPT-3.5 and GPT-4 as virtual patients noted a lack of nuanced emotional expression [10]. While our system received high general realism ratings, emotional realism was not specifically assessed as a separate domain in our evaluation. The high overall realism scores are encouraging, but a targeted evaluation of emotional realism and conversational nuance is warranted in future studies. Differences in realism ratings may also reflect advances in LLM capabilities and differences in prompt engineering approaches. Although the models we used were state-of-the-art at development time, they are already substantially outperformed by newer models [16], suggesting that results could further improve with updated models.

Our results confirm earlier studies such as Cook et al. [11], who developed a simpler LLM-based case simulator. We believe LLM-based simulators can provide a highly scalable, globally accessible, cost-effective tool for medical students and junior doctors to practise clinical reasoning across the full spectrum of clinical activity.

Regarding concerns about financial and infrastructure burdens of LLM-based simulations [17], our simulator can be adapted to work with any sufficiently capable LLM, including locally-hosted open-source models that could even be fine-tuned for this task. Currently, cloud-based inference remains most practical due to low latency and very low costs. However, for large institutions, local hosting may offer advantages in data privacy and potentially lower long-term costs despite upfront hardware investment.

Although our study has several strengths, it also has several limitations. Our evaluator sample size was small, limiting statistical power and generalisability. All evaluators were gastroenterology fellows from the hospital network of a single gastroenterology fellowship programme, which may limit generalisability to other settings. Also, although evaluator panellists were not involved in the development of the simulator, all evaluators are also co-authors of this manuscript, so we cannot rule out some degree of positivity bias. Evaluations relied on subjective Likert-scale ratings from a study-specific tool that has not been independently validated and we could not evaluate objective learner outcomes in this pilot study (e.g., pre/post knowledge tests, clinical reasoning assessments, or performance metrics), limiting definitive conclusions about educational effectiveness. Also, although open-ended free-text feedback was solicited, systematic qualitative data collection, such as structured interviews or thematic analysis, was not performed. Additional limitations include the lack of an automatic error-detection feedback system, as implemented in some previous studies [7, 18], which could enhance educational value. While our simulator includes simulated physical examination with finding interpretation, it cannot replace practising real physical examinations. The simulation is also primarily text/voice-based without images or video (e.g., endoscopy or radiology images) for interpretation. Additionally, while developed and pilot-evaluated for gastroenterology, the system could potentially be applied to other medical specialties, though this has not been evaluated and adjustments may be required.

## Conclusions

In conclusion, we developed and evaluated a clinical simulator enabling realistic simulation of entire gastroenterology clinical cases from history-taking to treatment and follow-up planning, fully generated by the system with high medical accuracy. These findings require validation in larger, multi-centre, multi-specialty studies incorporating objective learning outcomes. Future work should include evaluation by broader cohorts including medical students at varying training levels, application to other medical specialties, and extension to more complex interactions such as multidisciplinary discussions or real-time intra-procedural decision-making.

## Supplementary Information

Supplementary Material 1.

## Data Availability

Examples of full simulation transcripts and generated case reports are available in supplementary materials. Additional data are available upon reasonable request from the corresponding author.

## Abbreviations

GPT: Generative pre-trained transformer
LLM / LLMs: Large language model / large language models
N: Number
SD: Standard deviation
USMLE: United States Medical Licensing Examination
