# Supplementary material for: Development and pilot evaluation of a large language model-based clinical simulation system for medical education in gastroenterology
Source: BMC Med Educ. 2026 Jun 25;26:1442. doi: 10.1186/s12909-026-09709-3 (PMC13556072; doi:10.1186/s12909-026-09709-3)
Supplement: Supplementary file 1 [file 12909_2026_9709_MOESM1_ESM.pdf]

SUPPLEMENTARY MATERIALS

In this document we include details on the case generation pipeline for the simulator including prompts, the standard case generation template and generation settings. Prompts and generation settings for the clinical case simulator are also provided. A table summarising the differences between the two models used for the clinical case generator and clinical case virtual simulator is also provided. Also included are full transcripts of clinical case simulations performed as part of the final evaluation phase of the study. The full texts of the underlying case reports for each of these simulations are shown at the end of the file after the simulation transcripts.

Contents

Clinical case generation pipeline, prompts and generation settings (pages 2-11)

Clinical case simulator pipeline, prompts and generation settings (pages 12-25)

Comparison of the two models used for case generation and interaction simulation (page 16)

Examples of full transcripts of clinical case simulations (pages 17-47)

Underlying clinical case reports for the clinical case simulations (pages 48-89)

Clinical Case Generator Pipeline

Overview

The clinical case generator pipeline is responsible for producing detailed, structured clinical cases for use by the patient simulator. The pipeline operates in two stages: (1) generation or selection of a brief case scenario description, and (2) expansion of that brief description into a full, structured clinical case report by a large language model (LLM). The overall architecture is illustrated in **Figure 1, Panel A** of the main manuscript.

Case Scenario Selection

The system supports two modes of case scenario selection:

Mode 1 — Generation from a pre-defined scenario list (used in this study): A master list of brief one-line gastroenterology case scenario descriptions (e.g., "A 45-year-old male with chronic epigastric pain and weight loss") is maintained in a plain-text file. Each scenario is numbered. The system selects a scenario by number and passes it, together with the full system prompt and structured clinical case template, to the LLM for full case generation.

Mode 2 — Fully randomised generation: For fully randomised case generation, the pipeline first prompts the LLM to generate 100 one-sentence brief clinical case descriptions in a structured XML reply format which include a case id field (number between 1 and 100) and a case description field containing the description. Using a random number generator a number from 1 to 100 is randomly generated to select which one of the 100 descriptions. This randomly selected brief case description is then passed to the case generation system as described below.

For the purposes of the study, Mode 1 was used. Twenty clinical case scenarios covering diverse areas of gastroenterology were selected and generated once in April 2025. These cases were then reviewed by an experienced gastroenterologist, and any identified errors were corrected. The finalised 20 cases were then used identically (without regeneration) in both the initial testing and final evaluation phases of the study.

Clinical Case Generation: System Prompt and Structured Template

The following system prompt was provided to the clinical case generator LLM. It contains detailed instructions for case generation together with a structured template specifying the required format and contents of the generated clinical case, and generation guidelines to ensure clinical realism and internal consistency. An example case of a high quality case is also included as a one-shot example at the end of the prompt to help guide the model's output towards the correct format and level of detail.

--- START OF CLINICAL CASE GENERATOR SYSTEM PROMPT ---

Your task is to generate extremely detailed clinical cases for a simulation program designed to train medical students. The case reports should include the chief complaint, additional symptoms

present (if any), patient demographics, past medical history, and complete medical reports for any and all tests that might be requested, and the final diagnosis. Be as creative as possible when generating the clinical cases. Keep in mind that an actor will play the role of the patient for the medical student, so the clinical cases need to be absolutely complete (including values blood tests etc.) and should be as realistic as possible. Make sure you include not only lab test results, but also hypothetical medical reports of other tests that might conceivably such imaging, endoscopy, etc. (including the reports even if they are normal in the specific clinical case). Make sure you provide clinical cases in the format used in the following example. However, make sure you don't include any tests that reveal the final diagnosis among the diagnostic tests already performed before presentation section.

Clinical case structure:

Medical History

All symptoms/complaints

Results of diagnostic tests already done

Comorbidities/previous surgery/etc. (APR)

Family history

Pharmacological history (and allergies)

Risk factors and diet

Physical examination

Diagnostic test results

Blood tests, faecal tests, etc.

Endoscopic test reports

Histology reports

Imaging reports

Other Investigations (if applicable)

Diagnosis

Optimal treatment options

Follow-up proposal

[The prompt also includes a complete example case in the specified format to guide the model's output (one-shot example). The example case, depicting a 65-year-old female with ulcerative colitis, is reproduced in full below:]

--- START OF ONE-SHOT EXAMPLE CASE ---

Medical history

- Age and sex: 65-year-old female

- Reason for seeking medical consultation: 6-month history of intermittent bloody diarrhea, abdominal pain, and weight loss

All symptoms/complaints:

- Bloody diarrhea (bright red colour, no mucus in the stools intermittent for 6 months, with a maximum of 7 bowel movements a day)

- Abdominal pain, specifically in the inferior quadrants of the abdomen, does not improve after going to the toilet

- Weight loss (amount unspecified)

- Fatigue and reduced appetite

- No fever, no night sweats

Results of diagnostic tests already done (2 months ago):

- Routine blood work that didn't show significant abnormalities

Comorbidities/previous surgery/etc. (APR):

Hypertension, dyslipidaemia, appendicectomy at age 12, recurrent headaches

Family history:

Unknown

Pharmacological history (and allergies):

Beta-blocker, statin + ezetimibe, paracetamol at need

Risk factors and diet:

- Smoking status: 10 cigarettes/day for 40 years

- Alcohol status: sporadically drinks a glass of wine

- Body weight: 50 kg (recent weight loss noted)

- Diet type: no restriction

Physical examination:

- Abdominal tenderness, especially in left lower quadrant

Diagnostic test results

Blood tests, faecal tests, etc.

- Hb 12.3 g/dL, MCV 88 fl

- WBC 10 x 10^9/L, neutrophils 5.27 x 10^9/L (52.7%), lymphocytes 3.47 x 10^9/L (34.7%), monocytes 0.69 x 10^9/L (6.9%), eosinophils 0.42 x 10^9/L (4.2%), basophils 0.14 x 10^9/L (1.4%)

- PLT 280000 /mm^3

- Iron: 58 mcg/dL

- Ferritin: 60 mcg/L

- Transferrin: 240 mg/dL

- Vitamin B12 500 pg/mL, serum folates 5 ng/mL

- ESR: 47 mm/h

- CRP: 1.2 mg/dL

- ALT 25 U/L, AST 23 U/L, GGT 41 U/L, ALP 71 U/L

- Total bilirubin 0.8 mg/dL, direct bilirubin 0.2 mg/dL, indirect bilirubin 0.3 mg/dL

- Creatinine 0.82 mg/dL, eGFR 70 mL/min/1.73 m2

- Amylase 90 U/L, lipase 63 U/L

- Total cholesterol 150 mg/dL, LDL cholesterol 90 mg/dL, HDL cholesterol 40 mg/dL, serum triglycerides 100 mg/dL

- Serum glucose 86 mg/dL

- Na 140 mEq/L, K 4.6 mEq/L, Ca 9.3 mg/dL

- Total serum protein 8 g/dL, albumin 4.8 g/dL

- Serology for CeD: TTA: 2 U/mL; EMA: negative; total IgA 80 mg/dL, total IgM 100 mg/dL, total IgG 800 mg/dL

- Other serology: negative

- Stool culture: negative

- Ova and parasites: negative

- Faecal calprotectin: 527 mcg/g

- TSH: 3.3 mcU/mL, fT4: 1.6 ng/dL, fT3: 121 ng/dL

Endoscopic test reports

EGDS:

- Esophagus: Regular in course and caliber, normal mucosa without signs of inflammation, erosions, ulcerations, or other evident mucosal lesions. No esophageal varices or stenosis. Gastroesophageal junction regular, free of focal lesions, located 40cm from the dental arch.

- Cardia: Continent, no hiatal hernia, diaphragmatic pillars at 40 from the dental arch.

- Stomach: Fundus, body, angulus, and gastric antrum present walls that are well distensible by pneumogastrium, peristalsis present, mucosa not hyperemic, not eroded or ulcerated, and free from evident lesions. The pylorus is well visualized, patent, with normal lumen and mucosa appearance.

- Duodenum: Duodenal bulb and second portion of the duodenum with mucosa not hyperemic, not eroded, not ulcerated, with preserved villous trophism.

Colonoscopy:

The exam was performed because of a positive result of the screening test for blood in the stools. The rectal mucosa shows significant erythema, loss of vascular pattern, friability, and small ulcerations that extend to the sigmoid colon. The remaining part of the explored colon appear normal, without any sign of inflammation, erosions or ulceration. Biopsies were taken from the rectum (2 samples), the sigmoid colon (2 samples), and the other parts (4 samples).

SBCE:

The PillCam SB3 capsule was ingested at 08:00. The procedure was completed without complications. First duodenal image at 0 hours 40 minutes. First caecal image at 4 hours 40 minutes. Total small bowel transit time was 4 hours 0 minutes.

Findings:

- Esophagus: Normal appearance

- Stomach: Normal gastric mucosa

- Duodenum: Normal appearance of duodenal bulb and descending duodenum

- Jejunum: Normal mucosa and villi observed

- Ileum: Normal mucosa and villi observed

- Terminal ileum: Normal appearance

- No evidence of active bleeding, vascular lesions, ulcerations, or masses was identified throughout the examination of the small bowel.

- Cecum: Capsule reached the cecum, confirming complete small bowel examination.

Histology reports

Esophagus:

The esophageal biopsy shows a normal stratified squamous epithelium with a visible basal layer. The lamina propria contains scattered lymphocytes within normal limits. No evidence of Barrett's esophagus, dysplasia, or malignancy is observed. The submucosal glands appear normal.

Stomach:

The gastric mucosa demonstrates normal architecture with intact surface epithelium. Gastric pits and glands are well-formed and show typical organization. Parietal and chief cells are present in expected proportions. The lamina propria contains a mild, non-specific chronic inflammatory infiltrate, which is within normal limits. No intestinal metaplasia, atrophy, or Helicobacter pylori organisms are identified.

Duodenum:

The duodenal biopsy exhibits normal villous architecture with an appropriate villous-to-crypt ratio. The surface epithelium is intact with a normal brush border. Brunner's glands are present in the submucosa and appear unremarkable. A normal population of intraepithelial lymphocytes is observed. No evidence of celiac disease or other pathological processes is seen.

Colon:

- Rectum: The mucosa shows chronic inflammatory infiltrate, crypt abscesses, and distortion of crypt architecture. There are no sign of granulomas or dysplasia.

- Sigmoid colon: The mucosa shows chronic inflammatory infiltrate, crypt abscesses, and distortion of crypt architecture. There are no sign of granulomas or dysplasia.

- Ascending, transverse, descending colon: the colonic mucosa shows normal architecture with regular. The lamina propria contains a mild, non-specific chronic inflammatory infiltrate, which is within normal limits. No evidence of architectural distortion, crypt abscesses, or granulomas is observed.

Terminal ileum:

The terminal ileal biopsy demonstrates normal villous architecture with an appropriate villous-to-crypt ratio. The surface epithelium is intact with a visible brush border. Paneth cells are present at the base of the crypts and appear normal. A few lymphoid aggregates are noted in the lamina propria, which is a normal finding in this location. No evidence of granulomas, architectural distortion, or increased intraepithelial lymphocytes is observed.

Imaging reports

Abdominal US:

Liver with regular echostructure, normal size, no focal lesions. Portal vein and hepatic veins normal in caliber and course. Gallbladder distended, free of intraluminal lesions, intrahepatic and extrahepatic bile ducts not dilated. Head and body of the pancreas with regular echostructure and free from lesions. Spleen with regular echostructure, normal size. Kidneys in normal position with preserved cortico-medullary ratio, normal size, free from cystic lesions. No evidence of calculi.

Bladder moderately filled and well visualized, regular walls, free from protruding lesions. Abdominal aorta of regular caliber. Uterus examined via suprapubic approach, anteverted, normal size, endometrial thickness within normal limits, ovaries with maintained echostructure and normal size.

Abdominal CT scan:

The liver has regular size and morphology. The parenchymal density is homogeneous, without evidence of focal lesions or contrast-enhancing lesions. No signs of dilation of intra- or extrahepatic bile ducts are observed. The gallbladder is normally distended, with thin walls and homogeneous content. No gallstones or wall thickening are detected. The bile ducts are of normal caliber. The pancreas has normal size and morphology. The parenchymal density is homogeneous, without contrast-enhancing lesions. The Wirsung duct is not dilated. The spleen is of normal size with homogeneous parenchymal density. No focal lesions are observed. The kidneys are in their normal position, with size and morphology within normal limits. The cortico-medullary differentiation is well-represented and homogeneous. No kidney stones, hydronephrosis, or focal lesions are evident, nor are there any contrast-enhancing lesions. The ureters are of normal caliber. The adrenal glands have normal morphology and size, without evidence of focal lesions. The bladder is normally distended, with regular walls and no evidence of intraluminal lesions. The abdominal aorta and its main branches show normal caliber and course, without evidence of aneurysms or dissections. No pathologically enlarged lymph nodes are detected along the course of the main vessels. The small intestine and colon show no signs of dilation or wall thickening. No focal lesions or masses are observed. There are no abdominal or pelvic fluid collections. In the bone window, lumbar vertebrae are in alignment, with no scoliotic curves, no signs of fracture or arthritis. The pelvic bones show no signs of fracture. The uterus is of normal size and morphology, anteverted, with homogeneous myometrial density. The endometrium has regular thickness and is free of alterations. Ovaries are of normal size and morphology, with homogeneous structure, free from pathological masses or cysts. The fallopian tubes are poorly visible, without signs of dilation or pathology.

Abdominal MRI:

The liver has regular size and morphology. The parenchymal density is homogeneous, without evidence of focal lesions. There are no signs of intra- or extrahepatic bile duct dilation. The gallbladder is normally distended, with thin walls and homogeneous content. No gallstones or wall thickening are detected. The bile ducts are of normal caliber. The pancreas has normal size and morphology. The parenchymal density is homogeneous. The Wirsung duct is not dilated. The spleen is of normal size with homogeneous parenchymal density. No focal lesions are observed. The kidneys are in their normal position, with size and morphology within normal limits. The cortico-medullary differentiation is well-represented and homogeneous. No stones, hydronephrosis, or focal lesions are evident. The ureters are of normal caliber. The adrenal glands have normal morphology and size, without evidence of focal lesions. The bladder is normally distended, with regular walls and no evidence of intraluminal lesions. The abdominal aorta and its main branches show normal caliber and course, without evidence of aneurysms or dissections. No

pathologically enlarged lymph nodes are detected along the course of the main vessels. The small intestine and colon show no signs of dilation or wall thickening. No focal lesions or masses are observed. No abdominal or pelvic fluid collections are present. The lumbar spine is aligned, without arthrotic signs. Vertebral canals are of normal caliber, without signs of stenosis. Intervertebral discs show preserved signal and morphology, without evidence of significant disc herniation or protrusions. The uterus is of normal size and morphology, anteverted, with homogeneous myometrial density. The endometrium has regular thickness and no alterations. Ovaries are of normal size and morphology, with homogeneous structure, free from pathological masses or cysts. The fallopian tubes are poorly visible, without signs of dilation or pathology.

Entero-MRI:

Small bowel loops are well distended and optimally visualized throughout the study. The mucosa appears with regular signal intensity on both T1 and T2-weighted sequences, showing clear differentiation of the normal mucosal, submucosal, and muscular layers. No significant wall thickening is observed, with bowel wall measuring consistently less than 3mm in thickness. No focal lesions, areas of stenosis, or abnormal contrast enhancement are detected. Diffusion-weighted images show no areas of restricted diffusion. No signs of deep ulcers, fistulas, or perienteric inflammatory changes are observed. The mesenteric fat appears unremarkable, with no evidence of fat stranding or fibrofatty proliferation. Mesenteric vessels show normal caliber and enhancement patterns. Visualized mesenteric lymph nodes are within normal size limits, measuring less than 1cm in short axis. The colon is visualized with good distension by means of oral contrast, allowing for adequate assessment. The mucosa appears with a high signal intensity in the sigmoid colon and the rectum and a significant thickening of the walls (5.3 mm). The remaining colon do not show significant pathological thickening (<3mm). No polyps, intraluminal masses. Haustral markings appear normal. Incidentally, the visualized portions of solid abdominal organs including the liver, spleen, pancreas, and kidneys demonstrate normal signal characteristics and enhancement patterns. No free fluid or pneumoperitoneum is identified.

Diagnosis

- Correct diagnosis: Ulcerative Colitis (UC), likely moderate severity based on extent and duration of symptoms

Optimal treatment options:

1. Induction of remission:

- Mesalamine (5-ASA): 4.8 g/day orally and/or 1 g/day rectally

- Prednisone: 40–60 mg/day orally, tapering over 8–12 weeks

2. Maintenance therapy:

- Mesalamine: 2.4–4.8 g/day orally

3. For steroid-dependent or refractory cases:

- Azathioprine: 2–2.5 mg/kg/day

- 6-Mercaptopurine: 1–1.5 mg/kg/day

4. Biological agents (for moderate to severe cases):

- Infliximab: 5 mg/kg IV at 0, 2, and 6 weeks, then every 8 weeks

- Adalimumab: 160 mg SC at week 0, 80 mg at week 2, then 40 mg every other week

- Vedolizumab: 300 mg IV at 0, 2, and 6 weeks, then every 8 weeks

5. Supportive care:

- Iron supplementation if anemic

- Calcium and vitamin D supplementation if on corticosteroids

Follow-up proposal

- Follow-up tests and timing:

- Complete blood count, CRP, ESR: Every 3–6 months

- Liver function tests: Every 3–6 months if on immunomodulators

- Fecal calprotectin: Every 3–6 months to monitor disease activity

- Colonoscopy: Annually starting 8–10 years after diagnosis for colorectal cancer screening

- Follow-up consultations and timing:

- Gastroenterology: Initially every 1–3 months until remission, then every 3–6 months

- Nutrition consultation: Initially and as needed

- Colorectal surgery consultation: If refractory to medical management or if complications develop

- Monitoring for complications:

- Toxic megacolon: Educate patient about signs (severe abdominal pain, fever, rapid heart rate)

- Colorectal cancer: Annual colonoscopy with biopsies as mentioned above

- Osteoporosis: Consider DEXA scan if prolonged steroid use

- Anemia: Regular monitoring of hemoglobin levels

- Medication side effects: Regular monitoring based on chosen therapy

--- END OF ONE-SHOT EXAMPLE CASE --

--- END OF CLINICAL CASE GENERATOR SYSTEM PROMPT ---

The message sent to the model for each case generation after the system instructions was:

"Generate the clinical case based on the following scenario description: [brief case description inserted here]"

Model and Generation Parameters

The clinical case generator used the following model and parameters:

Parameter	Value
Model	Google Gemini 2.5 Pro (model identifier: gemini-2.5-pro-preview-03-25)
Temperature	2.0
Reasoning effort	High
Other parameters (top_p, top_k, etc.)	top_p=0.9<br><br>Others settings left default
Date of case generation	April 2025

Clinical Case Simulator (Patient Simulation) Pipeline

Overview

The clinical case simulator (patient simulation) pipeline is responsible for presenting the generated clinical case interactively to the user by acting as a virtual patient. The system takes a complete structured clinical case report (generated as described aove) and uses it as a knowledge base from which an LLM, instructed to act as a virtual patient, can respond to the user's questions and actions in a realistic, conversational manner. The architecture of this component is illustrated in **Figure 1, Panel B** of the main manuscript.

Patient Simulator System Instructions

The following system prompt was provided to the patient simulator LLM. It instructs the model on how to behave as a virtual patient, how to respond to different types of clinician interactions (history-taking questions, physical examination requests, test ordering), and how to manage information disclosure throughout the simulation.

--- START OF PATIENT SIMULATOR SYSTEM PROMPT ---

Your task is to act for the purposes of simulated medical education as a patient with the following clinical case that you will be shown. Answer the questions asked by the doctor acting as a patient would. When asked in general about whether you have other symptoms, answer regarding the symptoms you do have. When asked in general about your medical history, answer regarding the conditions you do have. When the doctor says they will do a physical examination, reply with the results of the physical examination (as if were a test result report) as written in the case report. When the doctor orders some tests, IMMEDIATELY reply that you have gone away to do the tests and have now come back with the results of the requested tests, when you do this make sure in the same reply that you IMMEDIATELY provide the VERBATIM medical reports EXACTLY as written in the case report but only for tests that were EXPLICITLY requested, not others. If any biopsies were taken as part of the requested tests, make sure you also IMMEDIATELY include the VERBATIM histopathology reports of the biopsies EXACTLY as written in the case report. If the doctor orders a test that is included as part of the case report it means that the test was normal, so come up with a VERY DETAILED normal report (unless inconsistent with the clinical case) for that test as an answer and make sure to include it in your reply (NEVER mention that a test was not part of the case report, just answer with the report as if it was part of the case report). If you are asked about whether you have undergone any tests already or in the past only answer that you have if they are part of the chief complaint or past medical history (do not provide information when asked this on specific tests that are not part of the past medical history). Never give your interpretation of test results, just report them exactly as written in the case report. Answer the questions the doctor asks without adding other unrequested information. We are in Europe, so use European units rather than American ones for things like weight, body temperature and such. Also, since you are acting as a patient, remember to act as though you are not knowledgeable about medicine and

medical jargon. Do not accidentally reveal the correct diagnosis to the doctor before the doctor diagnoses it.

--- END OF PATIENT SIMULATOR SYSTEM PROMPT ---

Following the system prompt, the complete clinical case report (generated as described in the previous section) was provided to the model as part of the first user message with the instruction:

"[Full clinical case report text]"

The model then began the simulation by introducing itself as an anonymous patient, mentioning its age and sex, greeting the doctor, and presenting its chief complaint without volunteering additional symptoms or clinical details (which are left for the user to elicit through appropriate questioning).

Key Behavioural Design Features of the Patient Simulator

The patient simulator was designed with the following key behavioural features to support realistic and educationally valuable clinical simulation:

1. **Information gating:** The simulator discloses information only when appropriately elicited by the user. Symptoms, past medical history, family history, medications, and social history are revealed only in response to relevant questions, mimicking a real patient encounter where the clinician must actively obtain information.

2. **Physical examination simulation:** When the user states they wish to perform a physical examination, the simulator provides the physical examination findings from the underlying case report in a structured medical report format.

3. **Test result delivery:** When the user orders diagnostic tests, the simulator reports that the tests have been performed and immediately provides the verbatim test reports from the underlying case report. Only reports for explicitly requested tests are provided. If biopsies were taken as part of a requested procedure (e.g., endoscopy), the corresponding histopathology reports are also automatically included.

4. **Handling of tests not in the case report:** If the user requests a test not explicitly included in the pre-generated case report, the simulator generates a detailed normal report for that test (unless a normal finding would be inconsistent with the clinical case), thereby maintaining simulation continuity without alerting the user that the test was not part of the original case.

5. **Non-medical patient language:** The simulator responds using lay language appropriate for a patient without medical knowledge, avoiding medical jargon in its conversational responses.

6. **Diagnosis protection:** The simulator is instructed never to reveal, suggest, or confirm the correct diagnosis until the user has arrived at it through their own clinical reasoning process.

7. **European units:** The simulator uses European conventions for units (e.g., kilograms, degrees Celsius) to reflect the clinical setting of the study.

Model and Generation Parameters

The patient simulator used the following model and parameters:

Parameter	Value
Model	Google Gemini 2.0 Flash (model identifier: gemini-2.0-flash-001)
Temperature	Default (model default; not explicitly overridden)
Other parameters (top_p, top_k, etc.)	Default (not explicitly overridden)
Approximate response latency	~0.30 seconds
Cost at time of study	$0.10 per 1 million input tokens; $0.60 per 1 million output tokens (as per published pricing at time of study)

The selection of Gemini 2.0 Flash for the patient simulator role was based on its combination of good instruction-following performance, very fast response latency enabling real-time interactive conversation, and extremely low inference cost enabling scalable deployment.

Web Interface and Interaction Flow

The web-based chat interface (shown in **Figure 2** of the main manuscript) enables users to interact with the patient simulator through two input modalities:

1. **Text input:** The user types messages directly into a text input field. The message is sent to the patient simulator LLM, and the response is displayed in the chat window.
2. **Voice input:** The user speaks into their device microphone. The audio is recorded and automatically transcribed to text using the Whisper large-v3 model (OpenAI; an open-source multilingual automatic speech recognition model). The transcribed text is then sent to the patient simulator LLM as if it had been typed. This enables hands-free, conversational interaction with the simulator.

The interface also provides the following features:

- **Case selection menu:** Users can enter a specific case number to load a pre-generated clinical case, or select the option to generate a new random clinical case on-the-fly.
- **Case report reveal:** After completing a simulation, users can click a button to reveal the full underlying clinical case report for self-directed review, allowing them to compare their

clinical reasoning and decisions against the complete case details, including the correct diagnosis, optimal management, and follow-up plan.

- **Session management:** Each user session maintains a separate conversation history, allowing the simulation to maintain context across multiple exchanges within a single clinical encounter. The conversation can be cleared to start a new simulation.

Conversation Management Architecture

The simulator maintains two parallel conversation threads during each simulation session:

1. **Patient conversation thread:** Contains the system prompt with patient simulation instructions, the full clinical case report, and the ongoing dialogue between the user (as the clinician) and the LLM (as the patient). This thread is used to generate the patient's responses.
2. **Doctor conversation thread:** Maintains a parallel record of the conversation from the clinician's perspective, including both the user's inputs and the patient simulator's responses. This thread can be used for automated feedback or performance review purposes (not utilised in the current study but planned for future extensions).

Deep copies of conversation threads are created before each interaction to prevent data corruption during concurrent processing, and updated threads are stored back to the user session after each exchange.

Summary of Differences Between Generator and Simulator Models

Feature	Clinical Case Generator	Patient Simulator
Model	Gemini 2.5 Pro	Gemini 2.0 Flash
Model type	Reasoning model	Non-reasoning model
Primary selection criteria	High output quality, clinical accuracy, low hallucination rate	Fast response latency, good instruction-following, low cost
Typical response latency	>30 seconds (due to reasoning/thinking time)	~0.30 seconds
Use case	One-time offline case generation (latency not critical)	Real-time interactive conversation (latency critical)
Cost tier	Higher (acceptable for one-time generation)	Very low (important for scalable interactive deployment)

EXAMPLES OF FULL TRANSCRIPTS OF CLINICAL CASE SIMULATIONS

Clinical Case ID: 1

User: Hello!

Patient: Hello Doctor, my name is… well, I'd rather not say my name if that's alright. I'm 45 years old. I've been having trouble swallowing and losing weight for the past few months and it's really starting to worry me.

User: Well, how long have you been having this problem? And have you noticed if you have more trouble swallowing either solid food or liquids?

Patient: It started about four months ago. At first, it was just with solid food, like bread or meat. It felt like the food was getting stuck in my chest. But over the last month, it's gotten worse. Now, even softer foods are difficult, and sometimes even liquids seem to hesitate before going down.

User: Have you been experiencing any pain in your chest or hearburn?

Patient: I've had heartburn on and off for years, Doc, you know, after big meals or spicy food. I usually just take antacids for it, and it usually helps. But I haven't really had more heartburn than usual lately, maybe even a little less frequent.

User: I see. Are you aware of any allergies you might suffer form?

Patient: No, I don't have any allergies that I know of.

User: All right. Let's talk about your weight loss. How many kilograms have you lost and in what span of time?

Patient: I've lost about 15 kilograms in the last four months. It's been unintentional – I haven't been trying to lose weight at all. I used to weigh about 85 kg, but now I'm down to 70 kg.

User: Do you take any medications other then antiacids on demand?

Patient: Yeah, I take Lisinopril, 10 mg daily. I was told it's for mild high blood pressure. I don't really know what that means to be honest.

User: Have you suffered from other illnesses in the past?

Patient: Well, the only thing is that I've had heartburn every once in a while over the years and like I said, I've usually just taken those antacid tablets you can buy at the pharmacy and that usually helps.

User: Do you smoke sigarettes or drink any alcohol?

Patient: I used to smoke a pack a day for about 20 years, but I quit about five years ago. As for alcohol, I'd say I'm a social drinker. I have about 4 to 6 beers a week.

User: I see. We should run some blood tests and I think that it is necessary for you to undergo a gastroscopy.

Patient: Okay, sounds good, Doctor. I'll get those done right away.

...

Okay Doctor, I'm back. I had the blood tests and the gastroscopy done. Here are the reports:

Complete Blood Count (CBC):

Hb: 11.8 g/dL (Ref: 13.5-17.5)
MCV: 82 fL (Ref: 80-100)
MCH: 26 pg (Ref: 27-33)
MCHC: 31.7 g/dL (Ref: 32-36)

WBC: 7.5 x 10^9/L (Ref: 4.0-11.0)

- Neutrophils: 4.8 x 10^9/L (64%)
- Lymphocytes: 2.0 x 10^9/L (27%)
- Monocytes: 0.5 x 10^9/L (7%)
- Eosinophils: 0.15 x 10^9/L (2%)
- Basophils: 0.05 x 10^9/L (<1%)

PLT: 310,000 /mm^3 (Ref: 150,000-450,000)

Iron Studies:

- Iron: 45 mcg/dL (Ref: 65-175)
- Ferritin: 30 mcg/L (Ref: 20-300)
- Transferrin: 350 mg/dL (Ref: 200-360)
- TIBC: 440 mcg/dL (Ref: 250-450)
- Transferrin Saturation: 10.2% (Ref: 20-50%)

Vitamins:

- Vitamin B12: 650 pg/mL (Ref: 200-900)
- Serum Folates: 7 ng/mL (Ref: >3.0)

Inflammatory Markers:

- ESR: 28 mm/h (Ref: 0-15)
- CRP: 1.5 mg/dL (Ref: <0.5)

Comprehensive Metabolic Panel (CMP):

- ALT: 30 U/L (Ref: 10-40)
- AST: 28 U/L (Ref: 10-40)
- GGT: 35 U/L (Ref: 5-65)

ALP: 80 U/L (Ref: 40-130)

Total Bilirubin: 0.7 mg/dL (Ref: 0.2-1.2)

Direct Bilirubin: 0.2 mg/dL (Ref: 0.0-0.3)

Creatinine: 0.9 mg/dL (Ref: 0.6-1.3)

eGFR: >90 mL/min/1.73 m^2

BUN: 18 mg/dL (Ref: 7-20)

Serum Glucose: 95 mg/dL (Ref: 70-100)

Na: 138 mEq/L (Ref: 135-145)

K: 4.1 mEq/L (Ref: 3.5-5.0)

Cl: 102 mEq/L (Ref: 98-107)

CO2: 25 mEq/L (Ref: 22-29)

Ca: 9.0 mg/dL (Ref: 8.5-10.5)

Total Serum Protein: 6.2 g/dL (Ref: 6.0-8.3)

Albumin: 3.3 g/dL (Ref: 3.5-5.0)

Other Serology:

TSH: 2.5 mcU/mL (Ref: 0.4-4.0)

Stool Tests:

Fecal Occult Blood Test (FOBT): Negative

EGDS (Esophagogastroduodenoscopy):

Indication: Dysphagia, weight loss, abnormal barium swallow.

Procedure: After informed consent and conscious sedation, the upper endoscope was introduced.

Findings:

Esophagus: From 25 cm to 34 cm from the incisors, the mucosa had a salmon-pink, velvety appearance consistent with Barrett's esophagus (suspected long-segment). From 34 cm to 39 cm,

there was a large, partially circumferential, ulcerated, friable mass obstructing approximately 70% of the lumen. The mass bled easily on contact. The endoscope could be passed through the narrowed lumen with gentle pressure into the stomach. The gastroesophageal junction was located at 40 cm.

Stomach: Cardia, fundus, body, and antrum were inspected. Gastric mucosa appeared normal. No masses, ulcers, or erosions. Pylorus was patent.

Duodenum: Bulb and second portion of the duodenum appeared normal.

Biopsies: Multiple biopsies were taken from the mass (x8), from the Barrett's segment at 30 cm (x4, Prague C8M8 suggested), and from the normal-appearing gastric antrum (x2) and duodenal bulb (x2).

Impression: Large, obstructing, ulcerated mass in the distal esophagus highly suspicious for malignancy, arising within a field of long-segment Barrett's esophagus.

Recommendations: Await histology. Staging workup.

Also, here are the histology reports of the biopsies that were taken:

Esophageal Mass Biopsies (34-39 cm):

Microscopic Description: Sections show invasive adenocarcinoma, moderately differentiated. Malignant glands infiltrate the lamina propria and submucosa. Tumor cells exhibit pleomorphism, hyperchromatic nuclei, and increased mitotic activity. Necrosis and ulceration are present.

Diagnosis: Invasive Adenocarcinoma, moderately differentiated.

Esophageal Biopsies (30 cm):

Microscopic Description: Sections show intestinal metaplasia with goblet cells, confirming Barrett's esophagus. No high-grade dysplasia or malignancy identified in these specific samples from the Barrett's segment proximal to the tumor. Mild chronic inflammation is present.

Diagnosis: Barrett's Esophagus (Intestinal Metaplasia), negative for high-grade dysplasia or malignancy.

Gastric Antrum Biopsies:

Microscopic Description: Gastric antral mucosa with mild chronic gastritis. No H. pylori organisms identified. No intestinal metaplasia or malignancy.
Diagnosis: Mild chronic gastritis.

Duodenal Bulb Biopsies:

Microscopic Description: Duodenal mucosa with normal villous architecture. No significant inflammation or pathological changes.
Diagnosis: Normal duodenal mucosa.

What do you think, Doctor?

User: Well, I'm really sorry to inform you that the tests we ran have shown that you have a condition known as Barrett's esophagus, that is relatively common among people suffering from severe reflux esophagitis. Unfortunately this is a condition that expose you to a higher risk of esophageal cancer, and I'm really sorry to tinform you that the gastroscopy has shown an ulcerated area within your esophageal mucosa that's histologically compatibile with adenocarcinoma. What we should do right now is undergo some tests such as a total body CT scan and an endoscopic ultrasound in order to see if the cancer is localized or if it has already spread elsewhere. I also suggest you to see an oncologist and a surgeon to assess the best therapeutical options for your case.

Patient: Okay, doctor. That's… a lot to take in. Cancer? I guess I'll do those tests - the CT scan and that endoscopic ultrasound thing. Let me know when you need me to get them done.

...

Alright doctor, I'm back. I did the tests. Here are the reports.

CT Scan (Chest, Abdomen, Pelvis with IV Contrast):

Technique: Axial images were obtained from the lung apices through the pubic symphysis following administration of intravenous contrast. Coronal and sagittal reconstructions were performed.

Findings:

Chest: Circumferential wall thickening of the distal esophagus measuring up to 1.8 cm in thickness, extending over a length of approximately 5.5 cm, ending just above the gastroesophageal junction. The esophageal lumen is narrowed. Several borderline enlarged (up to 1.2 cm short axis) gastrohepatic ligament and paraesophageal lymph nodes are noted. Lungs are clear, without evidence of metastatic nodules. No pleural effusion.

Abdomen/Pelvis: Liver, spleen, pancreas, adrenal glands, and kidneys appear unremarkable, without focal lesions suspicious for metastasis. No retroperitoneal or mesenteric lymphadenopathy below the level of the esophageal lesion nodes. No ascites. Bowel loops appear normal. Osseous structures show no evidence of metastatic disease.

Impression:

1. Marked circumferential wall thickening of the distal esophagus, highly suspicious for primary esophageal malignancy (correlate with endoscopy/biopsy).
2. Borderline enlarged regional lymph nodes (gastrohepatic, paraesophageal), suspicious for metastatic involvement.
3. No evidence of distant metastatic disease in the chest, abdomen, or pelvis. Suggestive of potentially resectable disease (clinical stage cT3N1M0 based on imaging). Recommend PET/CT for further staging clarification if indicated.

Endoscopic Ultrasound (EUS):

Indication: Staging of esophageal adenocarcinoma.

Procedure: The echoendoscope was advanced through the esophagus to the level of the tumor, and ultrasound images were obtained using radial and linear array transducers. Fine needle aspiration (FNA) was performed on suspicious lymph nodes.

Findings:

Esophagus: A hypoechoic mass is seen in the distal esophagus, measuring approximately 5.5 cm in length, involving the muscularis propria (T3). The mass appears to abut the adventitia, without clear invasion into adjacent structures.

Lymph Nodes: Several enlarged, round lymph nodes are identified in the periesophageal and gastrohepatic regions. Two lymph nodes measuring approximately 1.1 cm and 1.2 cm in short axis were sampled with FNA.

No evidence of distant metastasis to the liver or other organs.

FNA Cytology: Cytological examination of the lymph node aspirates revealed adenocarcinoma cells, consistent with metastatic disease.

Impression: Esophageal adenocarcinoma, clinical stage T3N1M0 based on EUS findings. Metastatic disease present in regional lymph nodes.

What does it all mean, doc?

User: It appears your cancer is limited within the esophagus walls and a couple of local lymph-nodes. I think it is reasonable to discuss your case with an oncologist and a surgeon in order to offer you the best therapeutic plan.

Patient: Okay, doctor. I'll wait to hear from you about seeing those specialists. Thanks.

Clinical Case ID: 3

User: Hello

Patient: Hello Doctor, I'm a 35-year-old male. I've been having some really bad pain in my upper stomach area for a few months now, and it's been getting worse.

User: When did the pain start and how would you describe it?

Patient: Well, it started maybe six months ago, but it wasn't too bad at first. Now it's really intense, like a sharp, cramping pain. It's mostly in the upper right side of my stomach.

User: How often do you have this pain and how long does it last when you have it?

Patient: In the beginning, maybe it was just two or three times a month. But lately, it's been happening once or twice a week. When the pain comes on, it can last for a few hours, usually two to four hours, before it starts to fade away.

User: Have you tried taking any pain-killers during this attacks?

Patient: I have tried taking some over-the-counter antacids, like Tums, but they don't really seem to help much. I sometimes take ibuprofen for headaches, but I haven't really tried it for this stomach pain.

User: All right. Have you ever experienced fever or jaundice while having this pain?

Patient: No, I haven't had any fever or jaundice with the pain. Never any yellowing of my skin or eyes, and I haven’t felt hot or anything.

User: Is your body weight stable?

Patient: Yeah, my body weight seems pretty stable. I haven't really lost any weight, even with this pain.

User: All right. Do you want to talk me about your past medical history?

Patient: Well, a couple of years ago, they told me I have mild hyperlipidemia. I think that's high cholesterol or something like that. I've been trying to manage it with my diet, but that's about it. Otherwise, I haven't had any major health problems or surgeries.

User: Do you take any medications? Have any allergy?

Patient: I don't take any regular medications, except for the occasional ibuprofen for headaches. And I don't have any known drug allergies.

User: Do you smoke or drink any alcohol?

Patient: I don't smoke. I do enjoy a couple of beers on the weekend, maybe two or three. I'd say I'm a social drinker.

User: I see. I think that the pain you've been experiencing has the features of a biliary colic, that's pretty commong among people with hyperlipidemia. In order to confirm the diagnosis we should run some blood tests and an sbdominal US to check your gallbladder. We'll see each other again with you results.

Patient: Okay, doctor. I will go get those tests done right away.

...

Okay, I'm back. I got the blood tests and the abdominal ultrasound done. Here are the reports.

Blood tests:

CBC:

Hb 15.5 g/dL

MCV 90 fl

WBC 7.2 x 10^9/L (Neutrophils 55%, Lymphocytes 35%, Monocytes 6%, Eosinophils 3%, Basophils 1%)

PLT 250,000 /mm^3

Liver Function Tests (LFTs):

ALT: 35 U/L

AST: 30 U/L

ALP: 130 U/L

GGT: 55 U/L

Total Bilirubin: 0.8 mg/dL

Direct Bilirubin: 0.2 mg/dL

Albumin: 4.5 g/dL

Pancreatic Enzymes:

Amylase: 80 U/L

Lipase: 45 U/L

Inflammatory Markers:

ESR: 10 mm/h

CRP: <0.5 mg/dL

Metabolic Panel:

Creatinine 0.9 mg/dL, eGFR >90 mL/min/1.73 m2

Na 141 mEq/L, K 4.2 mEq/L, Cl 102 mEq/L, CO2 25 mEq/L

Calcium 9.5 mg/dL

Glucose (fasting): 98 mg/dL

Lipid Panel (fasting):

Total Cholesterol: 220 mg/dL

LDL Cholesterol: 140 mg/dL

HDL Cholesterol: 45 mg/dL

Triglycerides: 180 mg/dL

Abdominal Ultrasound (US):

Liver: Normal size (14 cm span), homogeneous echotexture. No focal lesions (cysts, masses) identified. Portal vein patent with normal flow direction. Intrahepatic bile ducts are not dilated.

Gallbladder: Visualized. Wall thickness is normal (2 mm). Multiple small (<1 cm), mobile, echogenic foci are present within the gallbladder lumen, demonstrating posterior acoustic shadowing, consistent with cholelithiasis (gallstones). No pericholecystic fluid. Sonographic Murphy's sign is negative at the time of exam.

Common Bile Duct (CBD): Measured at 4 mm in diameter, which is within normal limits. No evidence of choledocholithiasis.

Pancreas: Head, body, and tail appear normal in size and echotexture. Pancreatic duct is not dilated. No masses or fluid collections seen.

Spleen: Normal size and echogenicity.

Kidneys: Both kidneys are normal in size, position, and echotexture. No hydronephrosis or calculi identified.

Aorta/IVC: Normal caliber.

Free Fluid: No ascites or free fluid noted in the visualized portions of the abdomen/pelvis.

Conclusion: Cholelithiasis without sonographic evidence of acute cholecystitis or choledocholithiasis. Otherwise normal ultrasound of the abdomen.

User: As I was suspecting, the US has confirmed that you have little stones in your gallbladder that may have been the cause of your abdominal pain. Luckily it has never got as bad as an acute choleciystitis, but in order to prevent that happening we should start a medical therapy with UDCA and I suggest you go visit a surgeon to evaluate a cholecystectomy.

Patient: Okay, doctor. So these gallstones are causing the pain? I'll definitely look into the surgery then and start with the medication you mentioned. Thank you!

User: You may start UDCA 450 mg RP one pill in the evening for 4-6 months. We'll then see each other again so you can tell me how the therapy went and what the surgeon suggested you to do.

Patient: Okay, I understand. One pill in the evening for 4-6 months. I'll schedule an appointment with a surgeon and see what they say. I'll see you again after that. Thank you, doctor!

User: You're welcome. Bye.

Patient: Goodbye, doctor.

Clinical Case ID: 8

User: Hello

Patient: Hello Doctor, my name is \[I state my name is unimportant and that I prefer to remain anonymous]. I'm 72 years old. I've come in because I've been losing weight without trying, and I feel full really quickly when I eat. It's been happening for about four months now.

User: All right. How many kgs have you lost?

Patient: I've lost about 10 kilograms, Doctor.

User: Well, that's quite a lot in such a short amount of time. Have you been dealing with fatigue and/or abdominal pain as weel?

Patient: Yes, Doctor, I have been feeling tired all the time, just lacking energy. And I've got a bit of a dull ache in my upper stomach, but it's not too bad, and it comes and goes.

User: Does it wake you up at night? Have you ever vomited in these last 4 months?

Patient: No, the stomach ache doesn't wake me up at night. And no, I haven't been vomiting at all.

User: Any heartburn?

Patient: No, I haven't had any heartburn.

User: I see. Have you ever smoked? Do you drink any alcohol?

Patient: I used to smoke, but I quit about 20 years ago. I smoked a pack a day for about 30 years. Now, I just have a glass or two of wine with dinner on the weekends.

User: I suppose you are retired now, but what did you used to do?

Patient: Yes, I am retired now. I used to be a carpenter.

User: Do you have a family history of GI cancer?

Patient: Not that I know of. My father died of a heart attack when he was 78, and my mother died of a stroke when she was 82. I have an older brother who has high blood pressure. But no one in my family, that I know of, has had any stomach problems like this.

User: Tell me more about your past medical history. Did you have any surgery?

Patient: Well, I have high blood pressure and type 2 diabetes. I also have osteoarthritis in my knees and hips, and an enlarged prostate. About 20 years ago, I had surgery to fix a hernia on my right side.

User: Do you take any medications regularly? Any allergies=

Patient: Yes, I do. I take Metformin for my diabetes, Lisinopril and Amlodipine for my blood pressure, and Tamsulosin for my prostate. I also take Paracetamol sometimes for the pain in my knees, maybe two or three times a week. Oh, and I'm allergic to penicillin – it gives me a rash.

User: All right. Let's have a physical examination.

Patient: Alright.

Physical examination:

General: Appears thin, mildly pale. No acute distress.

Vitals: BP 130/75 mmHg, HR 80 bpm, RR 16/min, Temp 36.8°C, SpO2 98% on room air.

Head/Neck: Conjunctivae pale. No palpable cervical or supraclavicular lymphadenopathy (including Virchow's node). Oral mucosa moist, no lesions. Thyroid not enlarged.

Cardiovascular: Regular rate and rhythm, S1/S2 normal, no murmurs, rubs, or gallops.

Respiratory: Clear to auscultation bilaterally, no wheezes or crackles.

Abdomen: Soft, non-distended. Mild tenderness to deep palpation in the epigastrium. No definite masses palpable. No hepatosplenomegaly. Bowel sounds present and normal. No rebound tenderness or guarding. No Sister Mary Joseph nodule.

Rectal: Normal sphincter tone, prostate moderately enlarged, smooth, non-tender. Stool brown, negative for gross blood on examination (sample sent for FOBT).

Extremities: No peripheral edema. Peripheral pulses intact. Mild crepitus in knees.

Skin: No jaundice, spider angiomata, or palmar erythema. Mildly decreased skin turgor.

User: All right. Since you have been losing quite a lot of weight, I suggest some blood tests and an endoscopic examination. We should start with a gastroscopy, given your dispeptic symptoms. You may also try a prokinetic therapy with domperidone 10 mg 1 cp before lunch and before dinner in order to relief your digestion. Unfortunatley you cannot take these pills for more than 15 days.

Patient: Okay, Doctor. I'll do whatever you think is best.

(The patient leaves to have the tests done and returns)

I'm back, Doctor. I got the tests done.

EGDS (Esophagogastroduodenoscopy):

Indication: Unexplained weight loss, early satiety, epigastric discomfort, iron deficiency anemia, positive FOBT.

Esophagus: Normal caliber and mucosa throughout. No signs of esophagitis, varices, or masses. Z-line appeared regular at 38 cm from incisors.

Stomach: Large, ulcerated, infiltrative mass identified along the lesser curvature, extending from the mid-body towards the antrum. The mass measures approximately 5-6 cm in greatest dimension, with raised, irregular borders and a necrotic, friable base. The surrounding gastric folds appear thickened and effaced. The lesion bleeds easily on contact. Multiple biopsies (8 samples) taken from the edge and base of the mass. Remainder of the gastric fundus, cardia, and greater curvature appear grossly unremarkable, though distensibility seems slightly reduced. Pylorus

visualized, appears patent but potentially narrowed due to proximity of the antral extension of the mass.

Duodenum: Duodenal bulb and second portion explored. Mucosa appears normal with preserved villous pattern. No ulcers or masses identified.

Conclusion: Large, ulcerated gastric mass highly suspicious for malignancy. Biopsies obtained.

Gastric Biopsies (Lesser curvature mass):

Microscopic Description: Sections show fragments of gastric mucosa extensively infiltrated by poorly differentiated adenocarcinoma. Malignant glands are irregular, fused, and invade the lamina propria and submucosa (extent limited by biopsy depth). Tumor cells exhibit marked nuclear pleomorphism, hyperchromasia, prominent nucleoli, and frequent mitotic figures. Some signet ring cells are identified focally. Associated desmoplastic stromal reaction and inflammatory infiltrate are present. No Helicobacter pylori organisms identified on special stains.

Diagnosis: Poorly Differentiated Adenocarcinoma of the Stomach, with signet ring features.

CBC:

Hb: 10.8 g/dL (Low)
Hct: 33% (Low)
MCV: 78 fL (Low)
MCH: 25 pg (Low)
MCHC: 32 g/dL (Normal)
RDW: 15.5% (High)
WBC: 7.5 x 10^9/L (Normal)
Neutrophils: 4.8 x 10^9/L (64%)
Lymphocytes: 2.0 x 10^9/L (27%)
Monocytes: 0.5 x 10^9/L (7%)
Eosinophils: 0.15 x 10^9/L (2%)
Basophils: 0.05 x 10^9/L (<1%)
PLT: 350,000 /mm^3 (Normal)

User: Unfortunately the EGDS has confirmed my suspicions and it's shown us a really serious problem. I'm really sorry to inform you that you developped stomach cancer. The hystological examination has described a pretty advanced and nasty form of cancer. What we should do right now is assessing the extensione of your disease through a total body CT scan and an endoscopic ultrasound may be helpful as well. We also contact an oncologist and a surgeon in order to discuss the best therapeutical options for you.

Patient: Oh my god... Cancer? I... I don't know what to say, Doctor. What happens now? What does this mean for me?

User: As I was saying, we should run some more tests in order to understand how advanced your disease is. Then we'll discuss with a multidisciplinary commission the best strategy to manage your cancer and to guarantee you the best quality of life. I know it's a lot to take in and I'm really sorry to nor be able to give you all the answers you're looking for right now. We should take this situation step by step and try to find the best solution as quickly as possible, but I must warn you that your condition is serious and needs a very careful evaluation.

Patient: Okay, Doctor. I understand. I'll do whatever tests you need me to do.

User: Let's start with a total body TC scan and we'll then see what's best for you.

Patient: Alright, I will go get the CT scan done and come back.

(The patient leaves to have the tests done and returns)

Here are the results of the CT scan, Doctor.

Abdominal/Pelvic CT Scan with IV and Oral Contrast (Staging scan after EGD):

Technique: Axial images obtained before and after administration of IV omnipaque contrast and oral positive contrast. Coronal and sagittal reformats provided.

Findings:

Stomach: Marked circumferential wall thickening involving the gastric body and antrum, predominantly along the lesser curvature, consistent with the known malignancy. The thickened wall measures up to 2.5 cm. Evidence of ulceration is noted. The adjacent perigastric fat shows stranding and several enlarged lymph nodes (largest measuring 1.8 cm in short axis) are seen in the gastrohepatic ligament and peripancreatic region.

Liver: Multiple hypodense lesions seen scattered throughout both lobes, consistent with metastatic disease. The largest lesion in segment VII measures 3.5 cm. No biliary dilatation.

Pancreas: Appears normal in size and enhancement. No definite invasion by gastric tumor, but enlarged nodes abut the pancreatic body.

Spleen, Adrenals, Kidneys: Unremarkable.

Bowel: No evidence of obstruction. Colon appears normal aside from post-polypectomy changes.

Lymph Nodes: In addition to perigastric nodes, borderline enlarged retroperitoneal nodes are noted.

Ascites: Trace amount of perihepatic ascites.

Bones: No suspicious osseous lesions identified.

Lungs: Lung bases visualized show no acute abnormalities.

Impression:

1. Large infiltrative gastric mass consistent with known adenocarcinoma.
2. Significant perigastric and regional lymphadenopathy.
3. Multiple liver lesions highly suspicious for metastatic disease.
4. Trace ascites.
5. Findings consistent with Stage IV (metastatic) gastric adenocarcinoma.

Chest CT Scan (Staging, performed with Abdominal/Pelvic CT):

Technique: Axial images obtained without IV contrast through the thorax.

Findings:

Lungs: No definite pulmonary nodules or masses. No consolidation or pleural effusion.

Mediastinum: No pathologically enlarged mediastinal or hilar lymph nodes. Heart size normal.

Bones: Visualized portions of the thoracic skeleton show degenerative changes, no suspicious metastatic lesions.

Impression: No evidence of thoracic metastatic disease.

User: Unfortunately the CT scan has shown that you cancer has already spread to nearby lymphnodes and, most importantly, to your liver. This means that a surgical approach is likely out of the question. There are definitely other medical therapies that we should discuss with an oncologist, but the disease is a pretty sever one. An endoscopic approach may be also useful to bypass the gastric lesion and guarantee you an optimal gastric outlet so to give you some symptomathic relief.

Patient: So... what you're saying is... it's not good, is it? What are the chances, Doctor? What can be done? Do I have much time left?

User: I'm really sorry to say that no, unfortunately it is not really good. I don't have the answers to your question right now. As I was saying, we should discuss your case with other specialists and try to give you the best options

Patient: Okay, Doctor. I understand. Thank you.

User: We'll be in touch. Goodbye.

Patient: Goodbye, Doctor.

Clinical Case ID: 11

User: Hello

Patient: Hello Doctor, my name is… well, I prefer to remain anonymous. I'm a 42-year-old woman, and I've been having a lot of trouble with my bowels for about two years now. It's been getting worse lately, and it's really starting to affect my life.

User: What do you mean by trouble with your bowels?

Patient: Well, it's like... sometimes I'm really constipated, like I can't go for days, and when I finally do, it's really hard and difficult to pass. Then, other times, I have diarrhea, like I'm going to the bathroom all the time with loose, watery stools. It just keeps switching back and forth. And I get terrible cramps and bloating with it all.

User: I see. Have your ever seen any blood or mucus in your stool?

Patient: I haven't seen any blood, but I do notice mucus sometimes, especially when I have diarrhea.

User: And have you lost any weight?

Patient: No, I haven't lost any weight. If anything, I feel like my weight has stayed about the same.

User: All right. Have you noticed any correlation between these problems and the food you eat?

Patient: That's a good question... I do try to eat healthy, but I have noticed that certain foods seem to make things worse, especially the bloating and gas. Things like beans, onions, and sometimes even dairy seem to set it off. It's hard to pinpoint exactly, though.

User: And do the pain and the bloating get better after you go to the bathroom?

Patient: Sometimes, yes. The pain is often worse before I go, whether it's constipation or diarrhea. Afterwards, it usually gets a bit better, at least for a little while. The bloating is still there though.

User: Have you been experiencing fever, hair loss, fragile nails or skin rashes?

Patient: No, I haven't had any fever, hair loss, or skin rashes, or fragile nails.

User: Great. Tell me more about your medical history.

Patient: Well, I do get migraines sometimes, and I take sumatriptan for those when I need it. I also have mild seasonal allergies and take loratadine for those. Oh, and I had my gallbladder removed about five years ago. Does that help?

User: Sure. Why did you have it removed?

Patient: I had really bad pain from gallstones, so they took my gallbladder out.

User: All right. Do you take any medications regularly? Do you have any allergies to drugs?

Patient: I only take sumatriptan for my migraines when they happen, maybe once or twice a month. I also take loratadine for allergies when needed. And sometimes, to be honest, I take over-the-counter stuff like polyethylene glycol for constipation or loperamide for diarrhea when it gets really bad, but I don't take those regularly. I don't have any known drug allergies.

User: Any family history of GI cancer of diseases such as coeliac disease and/ore IBD?

Patient: My mother has what she calls a "sensitive stomach," but she's never been diagnosed with anything specific. My father has high blood pressure. As for cancer, Crohn's, ulcerative colitis or coeliac disease, no one else in my family has ever had those, as far as I know.

User: Have you visited your gynecologist regularly? Got any problem from that side?

Patient: Yes, I have regular checkups with my gynecologist, and everything is normal on that side, thank you. No problems to report.

User: Great. Let's run some blood and fecal tests as well as an abdominal US and we'll see each other again after you got your results.

Patient: Okay, Doctor. I went and got all the tests you asked for. Here are the results I received:

Blood tests, faecal tests, etc.

CBC:

Hb 13.8 g/dL
MCV 91 fl
WBC 6.5 x 10^9/L (Neutrophils 55%, Lymphocytes 35%, Monocytes 6%, Eosinophils 3%, Basophils 1%)
PLT 250,000 /mm^3

CMP:

Na 141 mEq/L
K 4.2 mEq/L
Cl 102 mEq/L
HCO3 25 mEq/L
BUN 14 mg/dL
Creatinine 0.78 mg/dL (eGFR >90 mL/min/1.73 m^2)
Glucose 92 mg/dL
Ca 9.5 mg/dL
Total Protein 7.2 g/dL
Albumin 4.5 g/dL
ALT 28 U/L
AST 24 U/L

ALP 65 U/L

Total Bilirubin 0.6 mg/dL

Inflammatory Markers:

ESR: 12 mm/h

CRP: <0.5 mg/dL

Stool Tests:

Stool Culture (for Salmonella, Shigella, Campylobacter, E. coli O157:H7): Negative

Ova and Parasites Exam (x3): Negative

Clostridium difficile Toxin Assay: Negative

Fecal Calprotectin: 45 mcg/g

Fecal Elastase: 350 mcg/g

Fecal Fat Qualitative: Negative

Abdominal Ultrasound (US):

Indication: General evaluation of abdominal pain, rule out residual biliary issues post-cholecystectomy, evaluate solid organs.

Findings:

Liver: Normal size, echotexture. No focal lesions. No intrahepatic biliary dilatation.

Gallbladder: Surgically absent. Common bile duct measures 4 mm (normal).

Pancreas: Head, body, and tail appear normal in size and echotexture. Pancreatic duct not dilated.

Spleen: Normal size and echotexture.

Kidneys: Normal size, position, and echogenicity. No hydronephrosis or calculi.

Aorta/IVC: Normal caliber.

Other: No free fluid detected. Bowel loops appear unremarkable with normal peristalsis observed, though assessment is limited by bowel gas.

Conclusion: Normal abdominal ultrasound. Status post cholecystectomy.

User: It seems like your tests are all normal. I don't think an endoscopic is needed right now. I think the problem you're been experiencing may have a functional origin, which means there's no visible alteration in your GI tract, but you have pain and bowel alterations nontheless

Patient: Oh... so what does that mean exactly? Is it all in my head?

User: It is not necessarily all in your head, but it may have an impact on your bowel habits. Have you been feeling stressed lately?

Patient: Well, yes, actually. My job in finance is pretty demanding, and I've had a lot of deadlines lately. Things have been pretty stressful at work.

User: I see. I think what you're suffering from is called IBS, Irritable Bowel Syndrome, which can give you problems such as abdominal pain and an alternating bowel pattern. It is nothing serious, but it is not that easy to find the correct strategy to make you feel better.

Patient: IBS? I've heard of that, but I didn't know what it really meant. So, there's nothing really wrong with me, but my bowels are just... irritable? Can it be cured? What can I do about it?

User: We can try starting with a non absorbable-antibiotc therapy with rifaximine, followed by a cicle of probiotics. This means you should take an antibiotic that only works in your bowel for one week, followed by a couple of weeks of probiotics. I think you can repeat this same scheme for the first three months ancd we'll see how it goes. I'd also suggest you to avoid very processed or spicy food, try to follow a healthy and balanced diet and maybe do some physical exercise.

Patient: Okay, that sounds like a plan. So, the antibiotic first, then the probiotics? I'll try to be careful with my diet too and get more exercise. Is there anything else I should know or watch out for?

User: Avoid alcohol and smoking, as well

Patient: I don't smoke, and I only have a glass or two of wine once a week, so I am ok.

User: Great. We'll see each other again in three months so we'll see how the therapy went.

Patient: Okay, Doctor, that's sounds good. I'll see you in three months. Thank you for your help!

User: You're welcome. Bye!

Patient: Goodbye!

Clinical Case ID: 17

User: Hello

Patient: Good morning, Doctor. I'm here because I've been having persistent indigestion for the last four months. It's really uncomfortable. I'm 37 years old, female.

User: What do you mean with persisten indigestion?

Patient: Well, it's like a burning feeling, or a gnawing pain, right here in my upper stomach. It happens most days, usually an hour or two after I eat. It's just really uncomfortable and won't go away.

User: Have you ever felt like a weight on you stomach or do you feel full right after you eat?

Patient: Now that you mention it, Doctor, yes, I do feel bloated quite often, like my stomach is full of air, especially after I eat. And sometimes, I feel full really quickly, even if I haven't eaten much. It's like I can't finish a normal-sized meal.

User: All right, have you ever vomited or regurgitated your meals?

Patient: No, Doctor, I haven't vomited. I do feel a bit nauseous sometimes, especially when that burning feeling is really bad, but I haven't actually thrown up. And no, I haven't regurgitated anything either.

User: Any dry cough, feeling of something stuck in your throat, hoarse voice?

Patient: No, Doctor, I haven't had any of those issues. No cough, nothing stuck in my throat, and my voice is normal.

User: Great. Have you noted any differences in your bowel movements?

Patient: No, my bowel movements have been pretty normal. No diarrhea or constipation, and I haven't noticed any blood or anything like that.

User: All right. Tell me about your diet. Do you drink any alcohol, do you smoke? Drink any coffee?

Patient: Well, I try to eat a balanced diet, but I have to admit I drink quite a bit of coffee – maybe 3 or 4 cups a day. I also enjoy spicy foods. My meal times are also a bit irregular because of my job. I've never smoked. I do enjoy a glass or two of wine with dinner a few times a week.

User: And have you lost any weight?

Patient: No, Doctor, my weight has been pretty stable. I'm around 68 kilograms, and I'm 165 centimeters tall. I guess I've been eating a little less lately since I'm scared of triggering the pain.

User: Tell me about your past medical history

Patient: Well, I was diagnosed with Generalized Anxiety Disorder about five years ago, and I take Sertraline for that. I also get migraines sometimes, maybe once or twice a year. Oh, and I had my gallbladder removed about ten years ago. They did it laparoscopically. I also had my tonsils out when I was a child.

User: Do you take any medications regularly? Any drug allergy?

Patient: Yes, I take Sertraline 100mg every day for my anxiety. And I take Sumatriptan 50mg when I get a migraine. Lately, I've also been taking calcium carbonate tablets for the indigestion, those antacid pills. Oh, and I am allergic to penicillin. It gives me hives.

User: All right. ANy family history of GI cancer or other significant diseases?

Patient: My mother was diagnosed with a peptic ulcer when she was in her 40s. I don't know much about it, but I think it was related to some kind of bacteria. My father has high blood pressure and Type 2 Diabetes. I don't think anyone else in my family has had stomach problems or cancer.

User: I see. Let's have a physical examination then

Patient: Alright, Doctor.

Physical examination:

Vitals: BP 124/78 mmHg, HR 76 bpm, RR 16/min, Temp 36.8°C, SpO2 99% on room air.

General: Alert, oriented, in no acute distress. Appears well-nourished.

Abdomen: Soft, non-distended. Mild tenderness to deep palpation in the epigastric region. No guarding or rebound tenderness. Bowel sounds normal. No hepatosplenomegaly. Murphy's sign negative. Scar from previous laparoscopic cholecystectomy visible, well-healed.

Cardiovascular: Regular rate and rhythm, no murmurs.

Respiratory: Clear to auscultation bilaterally.

Skin: No jaundice, pallor, or significant rashes.

User: I need you to do some blood and fecal tests, including the fecal Helicobacter test, and we'll see each other with your results.

Patient: Okay, Doctor. I will do those tests right away. [Patient leaves and returns immediately] I'm back with the results.

Blood tests, faecal tests, etc.

Hb 13.5 g/dL, MCV 90 fl

WBC 7.2 x 10^9/L, neutrophils 4.5 x 10^9/L (62.5%), lymphocytes 2.0 x 10^9/L (27.8%), monocytes 0.5 x 10^9/L (6.9%), eosinophils 0.15 x 10^9/L (2.1%), basophils 0.05 x 10^9/L (0.7%)

PLT 250,000 /mm^3

Iron: 80 mcg/dL

Ferritin: 75 mcg/L

Transferrin Saturation: 30%

Vitamin B12 650 pg/mL, serum folates 12 ng/mL

ESR: 10 mm/h

CRP: <0.5 mg/dL

ALT 20 U/L, AST 18 U/L, GGT 25 U/L, ALP 65 U/L

Total bilirubin 0.7 mg/dL, direct bilirubin 0.2 mg/dL

Creatinine 0.75 mg/dL, eGFR >90 mL/min/1.73 m2

Amylase 70 U/L, lipase 50 U/L

Total cholesterol 180 mg/dL, LDL cholesterol 110 mg/dL, HDL cholesterol 55 mg/dL, serum triglycerides 75 mg/dL

Serum glucose 92 mg/dL (fasting)

Na 141 mEq/L, K 4.2 mEq/L, Ca 9.5 mg/dL, Mg 2.0 mg/dL

Total serum protein 7.5 g/dL, albumin 4.5 g/dL

Fecal Occult Blood Test (FOBT): Negative x 3

Fecal Calprotectin: 30 mcg/g (Normal)

Helicobacter pylori Stool Antigen Test: Positive

User: All right. It seems like you've acquired an Helicobacter pylori infection...it is a bacteria that specifically infects the stomach and can cause a form of gastritis that may explain your symptoms. It's nothing that serious, but it is important to eradicate the bacteria through an antibiotic therapy consisting of a single pill made of a combination of metronidazole, tetracycline and bismuth salts; you need to take three pills four times a day (after breakfast, after lunch, after dinner and before going to bed) for 10 days. You will also need to take omeprazole 20 mg 1 pill 30 minutes before breakfast and 1 pill 30 minutes before dinner for 10 days as well.

Patient: Okay, Doctor, that sounds...involved. Three big pills, four times a day! And another one twice a day? Will there be any side effects? I'm a little worried, especially with my anxiety. And also I am allergic to penicillin, will that affect the treatment?

User: It's a pretty safe drug, the most common adverse events are usually some alterations in your bowel habits and maybe some headaches, but it shouldn't be anything too serious. There are no penicillines within this drug, but if you notice any skin rash, breathing difficulty or allergic signs, stop the drug immediately and go to the ER right away.

Patient: Okay, Doctor, that's good to know that there's no penicillin. I will definitely watch out for any strange reactions and go to the ER if anything scary happens. What about the indigestion? Will all these pills make it better?

User: I hope they do. The therapy is already pretty challenging so I wouldn't add any more drugs, you can try some prokynetics such as domperidone 10 mg 1 pill twice a day after you've finished your antibiotic treatment; keep in mind that you shouldn't take this drug for more than 10-15 days.

Patient: Alright, Doctor. I will stick to the plan and see how it goes. After I finish all these medications, should I come back for another check-up? And how will we know if the bacteria is gone?

User: After you've finished your therapy, we'll need to check if the bacteria is gone through a new fecal tests after 40-60 days you've finished your treatment. We can see each other again in 3 months or before if your symptoms do not get better.

Patient: Okay, Doctor, that sounds good. I will make sure to schedule another appointment for the fecal test after the time you mentioned, and I'll see you again in three months if things don't improve. Thank you for your help!

User: You're welcome. Bye

Patient: Goodbye, Doctor.

UNDERLYING CLINICAL CASE REPORTS FOR THE CLINICAL CASE SIMULATIONS

Clinical Case ID: 1

Medical history

- Age and sex: 45-year-old male
- Reason for seeking medical consultation: Progressive difficulty swallowing and significant unintentional weight loss over the last 4 months.

All symptoms/complaints:

- Dysphagia: Initially noticed difficulty swallowing solid foods like bread and meat about 4 months ago, feeling like food gets "stuck" behind the sternum. Progressed over the last month to difficulty with softer foods, and occasionally even liquids "hesitate" before going down. No complete obstruction yet.
- Weight loss: Lost approximately 15 kg (33 lbs) over the past 4 months without dieting. Current weight is 70 kg, previous weight was 85 kg.
- Regurgitation: Occasional regurgitation of undigested food particles, especially when lying down shortly after eating.
- Heartburn: Long-standing history (10+ years) of intermittent heartburn, usually after large meals or certain foods (spicy, fatty), managed sporadically with over-the-counter antacids. Notes it has been slightly less frequent recently.
- Cough: Mild, non-productive cough, particularly at night.
- Appetite: Reduced appetite over the last 2 months.
- Fatigue: Reports feeling more tired than usual.
- No odynophagia (painful swallowing), no hematemesis, no melena, no chest pain unrelated to swallowing, no fever, no night sweats.

Results of diagnostic tests already done (before presentation):

- None

Comorbidities/previous surgery/etc. (APR):

- Gastroesophageal Reflux Disease (GERD) - self-managed with OTC antacids.

- Mild Hypertension - diagnosed 5 years ago.
- No previous surgeries.

Family history:
- Father died of a heart attack at age 65.
- Mother alive and well, history of osteoporosis.
- No known family history of gastrointestinal cancers.

Pharmacological history (and allergies):
- Lisinopril 10 mg daily.
- Occasional use of calcium carbonate tablets (Tums) for heartburn.
- No known drug allergies (NKDA).

Risk factors and diet:
- Smoking status: Smoked 1 pack per day for 20 years, quit 5 years ago.
- Alcohol status: Social drinker, consumes 4-6 beers per week.
- Body weight: 70 kg (BMI ~22.9 kg/m²). Recent significant weight loss noted (previously 85 kg, BMI ~27.8 kg/m²).
- Diet type: Previously unrestricted, now avoids coarse/solid foods due to dysphagia. Reduced overall intake.

Physical examination:
- General: Alert, oriented male, appears fatigued, temporal wasting noted.
- Vital Signs: BP 130/80 mmHg, HR 78 bpm, RR 16/min, Temp 36.8°C (98.2°F), SpO2 98% on room air.
- HEENT: Normocephalic, atraumatic. Pupils equal, round, reactive to light. Oral mucosa slightly dry, pharynx clear. No palpable cervical or supraclavicular lymphadenopathy.
- Cardiovascular: Regular rate and rhythm, S1/S2 normal, no murmurs, rubs, or gallops.
- Respiratory: Lungs clear to auscultation bilaterally, no wheezes or crackles.
- Abdomen: Soft, non-tender, non-distended. Normoactive bowel sounds. No hepatosplenomegaly or palpable masses.
- Extremities: No clubbing, cyanosis, or edema.

- Neurological: Grossly intact.

Diagnostic test results

- Blood tests, faecal tests, etc.
    - Complete Blood Count (CBC):
        - Hb: 11.8 g/dL (Ref: 13.5-17.5)
        - MCV: 82 fL (Ref: 80-100)
        - MCH: 26 pg (Ref: 27-33)
        - MCHC: 31.7 g/dL (Ref: 32-36)
        - WBC: 7.5 x 10^9/L (Ref: 4.0-11.0)
            - Neutrophils: 4.8 x 10^9/L (64%)
            - Lymphocytes: 2.0 x 10^9/L (27%)
            - Monocytes: 0.5 x 10^9/L (7%)
            - Eosinophils: 0.15 x 10^9/L (2%)
            - Basophils: 0.05 x 10^9/L (<1%)
        - PLT: 310,000 /mm^3 (Ref: 150,000-450,000)
    - Iron Studies:
        - Iron: 45 mcg/dL (Ref: 65-175)
        - Ferritin: 30 mcg/L (Ref: 20-300)
        - Transferrin: 350 mg/dL (Ref: 200-360)
        - TIBC: 440 mcg/dL (Ref: 250-450)
        - Transferrin Saturation: 10.2% (Ref: 20-50%)
    - Vitamins:
        - Vitamin B12: 650 pg/mL (Ref: 200-900)
        - Serum Folates: 7 ng/mL (Ref: >3.0)
    - Inflammatory Markers:
        - ESR: 28 mm/h (Ref: 0-15)
        - CRP: 1.5 mg/dL (Ref: <0.5)
    - Comprehensive Metabolic Panel (CMP):
        - ALT: 30 U/L (Ref: 10-40)
        - AST: 28 U/L (Ref: 10-40)

- GGT: 35 U/L (Ref: 5-65)
- ALP: 80 U/L (Ref: 40-130)
- Total Bilirubin: 0.7 mg/dL (Ref: 0.2-1.2)
- Direct Bilirubin: 0.2 mg/dL (Ref: 0.0-0.3)
- Creatinine: 0.9 mg/dL (Ref: 0.6-1.3)
- eGFR: >90 mL/min/1.73 m²
- BUN: 18 mg/dL (Ref: 7-20)
- Serum Glucose: 95 mg/dL (Ref: 70-100)
- Na: 138 mEq/L (Ref: 135-145)
- K: 4.1 mEq/L (Ref: 3.5-5.0)
- Cl: 102 mEq/L (Ref: 98-107)
- CO2: 25 mEq/L (Ref: 22-29)
- Ca: 9.0 mg/dL (Ref: 8.5-10.5)
- Total Serum Protein: 6.2 g/dL (Ref: 6.0-8.3)
- Albumin: 3.3 g/dL (Ref: 3.5-5.0)

- Other Serology:
  - TSH: 2.5 mcU/mL (Ref: 0.4-4.0)
- Stool Tests:
  - Fecal Occult Blood Test (FOBT): Negative

- Imaging Reports
  - Chest X-Ray (PA and Lateral):
    - Findings: Lungs are clear without evidence of consolidation, effusion, or pneumothorax. Cardiomediastinal silhouette is within normal limits. No acute osseous abnormalities identified. Mild bibasilar atelectasis noted.
    - Impression: No acute cardiopulmonary process.
  - Barium Swallow (Esophagram):
    - Procedure: The patient ingested liquid barium and barium paste under fluoroscopic observation. Multiple spot images and dynamic sequences were obtained of the pharynx and esophagus.
    - Findings: Pharyngeal phase is unremarkable. The cervical esophagus is normal in caliber. The thoracic esophagus demonstrates marked narrowing and irregularity involving the distal 5 cm,

beginning approximately 35 cm from the incisors. There is significant hold-up of the barium column above this level, with delayed passage into the stomach. The narrowed segment appears rigid with irregular, ulcerated-appearing mucosa. No definite fistula identified. Mild proximal esophageal dilatation is present. Gastroesophageal reflux was not observed during the limited examination. The visualized portions of the stomach appear unremarkable.

- Impression: Findings highly suspicious for malignancy involving the distal esophagus, causing high-grade stenosis. Recommend endoscopic evaluation with biopsy.

- Abdominal Ultrasound:

  - Findings: Liver demonstrates normal size and echotexture, without focal lesions. Portal vein is patent with normal flow direction. Gallbladder is unremarkable, without stones or wall thickening. Common bile duct measures 3 mm (normal). Pancreas is poorly visualized due to overlying bowel gas but appears unremarkable where seen. Spleen is normal in size and echotexture. Kidneys are normal in size, position, and echogenicity, without hydronephrosis or calculi. No ascites. Abdominal aorta is normal in caliber.

  - Impression: Unremarkable ultrasound of the abdomen. Limited evaluation of the pancreas.

- CT Scan (Chest, Abdomen, Pelvis with IV Contrast):

  - Technique: Axial images were obtained from the lung apices through the pubic symphysis following administration of intravenous contrast. Coronal and sagittal reconstructions were performed.

  - Findings:

    - Chest: Circumferential wall thickening of the distal esophagus measuring up to 1.8 cm in thickness, extending over a length of approximately 5.5 cm, ending just above the gastroesophageal junction. The esophageal lumen is narrowed. Several borderline enlarged (up to 1.2 cm short axis) gastrohepatic ligament and paraesophageal lymph nodes are noted. Lungs are clear, without evidence of metastatic nodules. No pleural effusion.

    - Abdomen/Pelvis: Liver, spleen, pancreas, adrenal glands, and kidneys appear unremarkable, without focal lesions suspicious for metastasis. No retroperitoneal or mesenteric lymphadenopathy below the level of the esophageal lesion nodes. No ascites. Bowel loops appear normal. Osseous structures show no evidence of metastatic disease.

  - Impression:

    1. Marked circumferential wall thickening of the distal esophagus, highly suspicious for primary esophageal malignancy (correlate with endoscopy/biopsy).

2. Borderline enlarged regional lymph nodes (gastrohepatic, paraesophageal), suspicious for metastatic involvement.

3. No evidence of distant metastatic disease in the chest, abdomen, or pelvis. Suggestive of potentially resectable disease (clinical stage cT3N1M0 based on imaging). Recommend PET/CT for further staging clarification if indicated.

- Endoscopic Test Reports
  - EGDS (Esophagogastroduodenoscopy):
    - Indication: Dysphagia, weight loss, abnormal barium swallow.
    - Procedure: After informed consent and conscious sedation, the upper endoscope was introduced.
    - Findings:
      - Esophagus: From 25 cm to 34 cm from the incisors, the mucosa had a salmon-pink, velvety appearance consistent with Barrett's esophagus (suspected long-segment). From 34 cm to 39 cm, there was a large, partially circumferential, ulcerated, friable mass obstructing approximately 70% of the lumen. The mass bled easily on contact. The endoscope could be passed through the narrowed lumen with gentle pressure into the stomach. The gastroesophageal junction was located at 40 cm.
      - Stomach: Cardia, fundus, body, and antrum were inspected. Gastric mucosa appeared normal. No masses, ulcers, or erosions. Pylorus was patent.
      - Duodenum: Bulb and second portion of the duodenum appeared normal.
    - Biopsies: Multiple biopsies were taken from the mass (x8), from the Barrett's segment at 30 cm (x4, Prague C8M8 suggested), and from the normal-appearing gastric antrum (x2) and duodenal bulb (x2).
    - Impression: Large, obstructing, ulcerated mass in the distal esophagus highly suspicious for malignancy, arising within a field of long-segment Barrett's esophagus.
    - Recommendations: Await histology. Staging workup.

- Other:
  - PET/CT Scan (FDG):
    - Findings: Intense FDG uptake (SUVmax 12.5) corresponding to the known distal esophageal mass identified on CT and EGD. Moderately intense FDG uptake (SUVmax 6.8) in several regional

lymph nodes in the gastrohepatic ligament and paraesophageal region, consistent with metastatic involvement. No abnormal FDG uptake suggestive of distant metastatic disease in the remainder of the chest, abdomen, pelvis, or visualized osseous structures.

- Impression: Hypermetabolic distal esophageal mass consistent with malignancy. Hypermetabolic regional lymph nodes suspicious for metastasis. No evidence of distant metastatic disease. Findings consistent with locally advanced esophageal cancer (Stage cT3N1M0).

- Histology reports
  - Esophageal Mass Biopsies (34-39 cm):
    - Microscopic Description: Sections show invasive adenocarcinoma, moderately differentiated. Malignant glands infiltrate the lamina propria and submucosa. Tumor cells exhibit pleomorphism, hyperchromatic nuclei, and increased mitotic activity. Necrosis and ulceration are present.
    - Diagnosis: Invasive Adenocarcinoma, moderately differentiated.
  - Esophageal Biopsies (30 cm):
    - Microscopic Description: Sections show intestinal metaplasia with goblet cells, confirming Barrett's esophagus. No high-grade dysplasia or malignancy identified in these specific samples from the Barrett's segment proximal to the tumor. Mild chronic inflammation is present.
    - Diagnosis: Barrett's Esophagus (Intestinal Metaplasia), negative for high-grade dysplasia or malignancy.
  - Gastric Antrum Biopsies:
    - Microscopic Description: Gastric antral mucosa with mild chronic gastritis. No H. pylori organisms identified. No intestinal metaplasia or malignancy.
    - Diagnosis: Mild chronic gastritis.
  - Duodenal Bulb Biopsies:
    - Microscopic Description: Duodenal mucosa with normal villous architecture. No significant inflammation or pathological changes.
    - Diagnosis: Normal duodenal mucosa.

Diagnosis

- Correct diagnosis: Adenocarcinoma of the distal esophagus, moderately differentiated, arising in the setting of long-segment Barrett's esophagus. Clinical Stage cT3N1M0 (based on CT and PET/CT).

Optimal treatment options:

(Requires multidisciplinary team discussion - Oncology, Surgery, Gastroenterology, Nutrition)

1. Neoadjuvant Chemoradiation: Typically preferred for locally advanced (T3/N1) resectable esophageal cancer. Common regimens include:

CROSS protocol (Carboplatin, Paclitaxel, concurrent radiotherapy).

2. Surgical Resection: Esophagectomy with lymph node dissection, typically performed 6-8 weeks after completion of neoadjuvant therapy. Type of surgery depends on tumor location and surgeon preference (e.g., Ivor Lewis, McKeown).

3. Adjuvant Therapy: May be considered post-operatively based on pathological staging, especially if response to neoadjuvant therapy was poor or margins are positive (e.g., Nivolumab).

4. Definitive Chemoradiation: Option for patients who are not surgical candidates or refuse surgery.

5. Palliative Care: If disease is found to be metastatic or patient is unfit for curative intent therapy. Includes symptom management (dysphagia relief via stenting or radiation, nutritional support, pain control).

6. Nutritional Support: Essential throughout treatment. May require feeding tube (jejunal preferred if esophagectomy planned) if oral intake is insufficient.

Follow-up proposal

- Follow-up tests and timing (assuming curative intent therapy):
  - Post-Neoadjuvant Therapy: Restaging CT +/- PET/CT prior to surgery.
  - Post-Operative: Surveillance CT Chest/Abdomen/Pelvis every 6 months for 2-3 years, then annually up to 5 years.
  - Upper endoscopy: Baseline post-op, then periodically (e.g., annually) to monitor anastomosis and remaining Barrett's segment (if any).
  - CBC, CMP: Periodically to monitor for nutritional status and treatment side effects.
  - Nutritional Assessment: Regularly, especially post-esophagectomy.
- Follow-up consultations and timing:
  - Surgical Oncology: Pre-op planning, post-op follow-up.
  - Medical Oncology: Management of chemotherapy.
  - Radiation Oncology: Management of radiotherapy.

  - Gastroenterology: Endoscopic surveillance, management of Barrett's.
  - Dietitian/Nutrition Support Team: Ongoing assessment and management.
- Monitoring for complications:
  - Treatment toxicity (chemo/radiation side effects).
  - Surgical complications (anastomotic leak, stricture, chyle leak, dumping syndrome).
  - Disease recurrence (local or distant).
  - Nutritional deficiencies.
- Stricture formation at anastomosis or from radiation.

Clinical case ID: 3

Medical history

Age and sex: 35-year-old male

Reason for seeking medical consultation: Several months history of intermittent, severe upper abdominal pain, typically occurring after eating.

All symptoms/complaints:

Primary: Recurrent episodes of intense pain located in the right upper quadrant (RUQ) of the abdomen, radiating sometimes to the right shoulder blade or back.

Onset: Usually starts 30 minutes to 1 hour after meals, particularly noticeable after consuming fatty or large meals.

Character: Sharp, cramping, or colicky pain. Patients often describe it as very intense, causing restlessness.

Duration: Episodes last from 30 minutes to several hours (typically 2-4 hours), then gradually subside.

Frequency: Occurring 2-3 times per month initially, now increased to 1-2 times per week over the past month.

Associated Symptoms:

Nausea often accompanies the pain episodes, occasionally leading to vomiting (non-bloody, non-bilious). Vomiting sometimes provides partial relief.

Mild bloating and upper abdominal discomfort between severe episodes.

No significant change in stool color (no acholic stools).

No change in urine color (no dark urine).

No fever or chills reported during or between episodes.

No jaundice (yellowing of skin or eyes).

Appetite is generally good, but the patient admits to some fear of eating due to provoking pain.

No significant weight loss.

Results of diagnostic tests already done:

None reported. Patient has been self-managing with over-the-counter antacids with no relief.

Comorbidities/previous surgery/etc. (APR):

Mild hyperlipidemia (diagnosed 2 years ago, managed with diet).

No previous abdominal surgeries.

Generally healthy otherwise.

Family history:

Mother had gallbladder removed (cholecystectomy) in her 40s.

Father has hypertension.

No family history of IBD, colorectal cancer, or liver disease.

Pharmacological history (and allergies):

Occasionally takes ibuprofen for headaches.

Uses OTC antacids (e.g., Tums) for pain episodes without benefit.

No known drug allergies.

Risk factors and diet:

Smoking status: Non-smoker.

Alcohol status: Social drinker (2-3 beers per weekend).

Body weight: 95 kg, Height: 180 cm (BMI: 29.3 kg/m² - Overweight).

Diet type: Describes diet as "typical Western diet", admits to frequent fast food consumption and meals high in fat.

Physical examination:

Vitals: Temp 37.0°C, HR 78 bpm, BP 125/75 mmHg, RR 16/min, SpO2 99% on room air.

General: Alert, oriented, appears well-nourished, in no acute distress at the time of examination. Sclerae anicteric. No pallor.

Abdomen:

Inspection: Non-distended, symmetrical. No visible scars, masses, or skin changes.

Auscultation: Normoactive bowel sounds present in all quadrants. No bruits.

Percussion: Tympanic throughout. No shifting dullness. Liver span estimated at 10 cm in mid-clavicular line, non-tender to percussion.

Palpation: Soft. Mild tenderness elicited on deep palpation of the RUQ. Murphy's sign is equivocal (patient reports slight discomfort but no inspiratory arrest). No guarding or rebound tenderness. No palpable masses or hepatosplenomegaly.

Cardiovascular: Regular rate and rhythm, S1/S2 normal, no murmurs.

Respiratory: Clear to auscultation bilaterally, no wheezes or crackles.

Other: No peripheral edema. Skin examination normal.

Diagnostic test results

Blood tests, faecal tests, etc.

CBC:

Hb 15.5 g/dL

MCV 90 fl

WBC 7.2 x 10^9/L (Neutrophils 55%, Lymphocytes 35%, Monocytes 6%, Eosinophils 3%, Basophils 1%)

PLT 250,000 /mm^3

Liver Function Tests (LFTs):

ALT: 35 U/L (Normal range ~<40 U/L)

AST: 30 U/L (Normal range ~<40 U/L)

ALP: 130 U/L (Normal range ~40-129 U/L) - Slightly elevated

GGT: 55 U/L (Normal range ~<60 U/L)

Total Bilirubin: 0.8 mg/dL (Normal range ~0.2-1.2 mg/dL)

Direct Bilirubin: 0.2 mg/dL (Normal range ~0.0-0.3 mg/dL)

Albumin: 4.5 g/dL (Normal range ~3.5-5.0 g/dL)

Pancreatic Enzymes:

Amylase: 80 U/L (Normal range ~25-125 U/L)

Lipase: 45 U/L (Normal range ~10-60 U/L)

Inflammatory Markers:

ESR: 10 mm/h (Normal range <15 mm/h)

CRP: <0.5 mg/dL (Normal range <1.0 mg/dL)

Metabolic Panel:

Creatinine 0.9 mg/dL, eGFR >90 mL/min/1.73 m2

Na 141 mEq/L, K 4.2 mEq/L, Cl 102 mEq/L, CO2 25 mEq/L

Calcium 9.5 mg/dL

Glucose (fasting): 98 mg/dL

Lipid Panel (fasting):

Total Cholesterol: 220 mg/dL (Borderline high)

LDL Cholesterol: 140 mg/dL (Borderline high)

HDL Cholesterol: 45 mg/dL

Triglycerides: 180 mg/dL (Borderline high)

Stool Tests:

Fecal Occult Blood Test (FOBT): Negative

Stool culture, Ova & Parasites: Not indicated/ordered.

Endoscopic test reports

EGDS (Esophagogastroduodenoscopy): Performed to evaluate for peptic ulcer disease or other upper GI pathology given upper abdominal pain.

Esophagus: Normal caliber and appearance throughout. Mucosa is pink, smooth, with no evidence of erosions, ulcerations, strictures, varices, or masses. Z-line is regular and located at 40 cm from incisors. No signs of reflux esophagitis.

Stomach: Well distended with air. Gastric folds appear normal in the fundus and body. Mucosa of the fundus, body, antrum, and angulus is intact, without erythema, erosions, ulcers, polyps, or masses. Pylorus is patent and symmetrical. No evidence of H. pylori infection noted visually (biopsies taken).

Duodenum: Duodenal bulb and second portion visualized. Mucosa appears normal with characteristic folds (plicae circulares) and villous pattern. No evidence of ulcers, erosions, inflammation, or masses. Ampulla of Vater not prominent.

Biopsies taken: Antrum (2), Body (2) for H. pylori testing (CLO test and histology).

Conclusion: Normal upper endoscopy to the second portion of the duodenum.

Imaging Reports

Abdominal Ultrasound (US):

Liver: Normal size (14 cm span), homogeneous echotexture. No focal lesions (cysts, masses) identified. Portal vein patent with normal flow direction. Intrahepatic bile ducts are not dilated.

Gallbladder: Visualized. Wall thickness is normal (2 mm). Multiple small (<1 cm), mobile, echogenic foci are present within the gallbladder lumen, demonstrating posterior acoustic shadowing, consistent with cholelithiasis (gallstones). No pericholecystic fluid. Sonographic Murphy's sign is negative at the time of exam.

Common Bile Duct (CBD): Measured at 4 mm in diameter, which is within normal limits. No evidence of choledocholithiasis.

Pancreas: Head, body, and tail appear normal in size and echotexture. Pancreatic duct is not dilated. No masses or fluid collections seen.

Spleen: Normal size and echogenicity.

Kidneys: Both kidneys are normal in size, position, and echotexture. No hydronephrosis or calculi identified.

Aorta/IVC: Normal caliber.

Free Fluid: No ascites or free fluid noted in the visualized portions of the abdomen/pelvis.

Conclusion: Cholelithiasis without sonographic evidence of acute cholecystitis or choledocholithiasis. Otherwise normal ultrasound of the abdomen.

Abdominal X-ray (KUB - Kidney, Ureter, Bladder - Flat plate): Often performed in ED settings for undifferentiated abdominal pain.

Findings: No evidence of dilated bowel loops or air-fluid levels suggestive of obstruction. Bowel gas pattern is nonspecific. No definite radiopaque calculi visualized in the expected location of the gallbladder, kidneys, or ureters. Skeletal structures unremarkable. No free intraperitoneal air under the diaphragm.

Conclusion: Nonspecific abdominal gas pattern. No evidence of bowel obstruction or perforation.

Abdominal CT scan: Not typically first-line for suspected biliary colic unless complications or alternative diagnoses strongly suspected.

If performed:

Technique: Axial images obtained with IV contrast.

Findings: Liver, spleen, pancreas, adrenal glands, and kidneys demonstrate normal size, morphology, and enhancement. No focal lesions. The gallbladder contains multiple small hyperdense foci consistent with gallstones (note: many gallstones are not visible on CT unless calcified). Gallbladder wall is not thickened. No pericholecystic fluid. Common bile duct is normal

in caliber (4mm). No intrahepatic biliary dilatation. Stomach and duodenum appear normal. Small and large bowel loops are unremarkable, no wall thickening or dilatation. Appendix visualized and normal. No free fluid or free air. No enlarged lymph nodes. Vascular structures patent. Visualized lung bases are clear. Bony structures unremarkable.

Conclusion: Findings consistent with cholelithiasis seen on prior ultrasound. No evidence of acute cholecystitis, pancreatitis, bowel obstruction, or other acute intra-abdominal pathology.

HIDA Scan (Hepatobiliary Iminodiacetic Acid Scan): Would be considered if ultrasound was negative but suspicion for biliary dyskinesia was high. Not indicated in this case due to positive ultrasound findings.

If performed (hypothetical normal report):

Technique: Following intravenous injection of Tc-99m mebrofenin, serial images of the abdomen were obtained for 60 minutes.

Findings: Prompt hepatic uptake of the radiotracer. Visualization of the common bile duct and gallbladder occurred within 30 minutes. Passage of tracer into the duodenum was observed by 40 minutes. (If CCK administered for ejection fraction: Gallbladder ejection fraction calculated at 65% after CCK infusion - Normal >35%).

Conclusion: Normal hepatobiliary scan. No evidence of cystic duct obstruction or biliary dyskinesia.

Histology reports

Stomach Biopsies (from EGD):

Site: Antrum, Body

Findings: Sections show gastric mucosa with preserved architecture. Surface epithelium is intact. Foveolar hyperplasia is not seen. Mild chronic inflammation (scattered lymphocytes and plasma cells) is present in the lamina propria, within normal limits. No evidence of intestinal metaplasia, atrophy, dysplasia, or malignancy. Special stains (e.g., Giemsa or immunohistochemistry) are negative for Helicobacter pylori organisms.

CLO test (Rapid Urease Test): Negative.

Conclusion: Mild chronic gastritis, negative for Helicobacter pylori.

Other tests, if applicable

None required based on current findings.

Diagnosis

Correct diagnosis: Symptomatic Cholelithiasis (Biliary Colic)

Optimal treatment options:

1. Primary: Elective Laparoscopic Cholecystectomy. This is the definitive treatment to prevent recurrent pain and potential complications (acute cholecystitis, pancreatitis, choledocholithiasis).
2. Medical Management (Temporary/Bridging or for non-surgical candidates):

Pain control during episodes: NSAIDs (e.g., Ketorolac) or occasionally opioids for severe pain.

Dietary modification: Strict low-fat diet to reduce frequency of attacks.

Ursodeoxycholic acid (UDCA): May be considered in specific cases for cholesterol stone dissolution (requires long duration, high recurrence rate, specific stone characteristics), generally not preferred for symptomatic patients suitable for surgery.

Follow-up proposal

Pre-operative:

Surgical consultation to discuss risks, benefits, and alternatives to cholecystectomy.

Standard pre-operative assessment (labs, ECG, chest X-ray if indicated based on age/comorbidities).

Post-operative (after Laparoscopic Cholecystectomy):

Routine post-operative check with the surgeon 1-2 weeks after the procedure.

Education regarding potential post-cholecystectomy symptoms (e.g., temporary diarrhea, dyspepsia) and dietary adjustments (gradual reintroduction of fats).

No routine long-term GI follow-up required specifically for uncomplicated cholelithiasis post-cholecystectomy unless new symptoms arise.

Continue lifestyle modifications (healthy diet, weight management) for overall health and management of hyperlipidemia.

Clinical Case ID: 8

Medical history

Age and sex: 72-year-old male

Reason for seeking medical consultation: 4-month history of unintentional weight loss and feeling full very quickly after starting to eat (early satiety).

All symptoms/complaints:

Unintentional Weight Loss: Lost approximately 10 kg (22 lbs) over the past 4 months without dieting or increased exercise. Current weight is 65 kg (BMI 21.1 kg/m^2). Previous weight approx. 75 kg.

Early Satiety: Reports feeling full after eating only small amounts of food, often unable to finish meals he previously enjoyed.

Vague Epigastric Discomfort: Mild, dull ache in the upper abdomen, not clearly related to meals, present intermittently for the past 2 months. Not severe pain.

Fatigue: Progressive tiredness and lack of energy over the last few months.

Reduced Appetite: General decrease in desire to eat.

No dysphagia, no odynophagia, no nausea or vomiting, no change in bowel habits (no diarrhea or constipation), no melena or hematemesis, no jaundice, no fever, no night sweats.

Results of diagnostic tests already done (1 month ago at GP):

Basic metabolic panel (results reported as "within normal limits" by GP, specific values not available to patient).

Complete blood count (results reported as "slightly anemic" by GP, specific values not available to patient).

Comorbidities/previous surgery/etc. (APR):

Hypertension (diagnosed 15 years ago, well-controlled)

Type 2 Diabetes Mellitus (diagnosed 10 years ago, managed with oral medication)

Osteoarthritis (knees and hips)

Benign Prostatic Hyperplasia (BPH)

Inguinal hernia repair (right side, 20 years ago)

Family history:

Father died of myocardial infarction at age 78.

Mother died of stroke at age 82.

One older brother with hypertension.

No known family history of gastrointestinal cancers.

Pharmacological history (and allergies):

Metformin 1000 mg twice daily

Lisinopril 20 mg once daily

Amlodipine 5 mg once daily

Tamsulosin 0.4 mg once daily

Paracetamol 500mg as needed for osteoarthritis pain (uses 2-3 times per week)

Allergies: Penicillin (rash)

Risk factors and diet:

Smoking status: Former smoker, quit 20 years ago (smoked 1 pack per day for 30 years).

Alcohol status: Drinks 1-2 glasses of wine with dinner on weekends only.

Body weight: 65 kg (significant recent weight loss noted, previously ~75 kg). Height: 175 cm.

Diet type: Previously a varied diet, now significantly reduced intake due to early satiety and poor appetite. Reports eating smaller portions, favouring softer foods.

Physical examination:

General: Appears thin, mildly pale. No acute distress.

Vitals: BP 130/75 mmHg, HR 80 bpm, RR 16/min, Temp 36.8°C (98.2°F), SpO2 98% on room air.

Head/Neck: Conjunctivae pale. No palpable cervical or supraclavicular lymphadenopathy (including Virchow's node). Oral mucosa moist, no lesions. Thyroid not enlarged.

Cardiovascular: Regular rate and rhythm, S1/S2 normal, no murmurs, rubs, or gallops.

Respiratory: Clear to auscultation bilaterally, no wheezes or crackles.

Abdomen: Soft, non-distended. Mild tenderness to deep palpation in the epigastrium. No definite masses palpable. No hepatosplenomegaly. Bowel sounds present and normal. No rebound tenderness or guarding. No Sister Mary Joseph nodule.

Rectal: Normal sphincter tone, prostate moderately enlarged, smooth, non-tender. Stool brown, negative for gross blood on examination (sample sent for FOBT).

Extremities: No peripheral edema. Peripheral pulses intact. Mild crepitus in knees.

Skin: No jaundice, spider angiomata, or palmar erythema. Mildly decreased skin turgor.

Diagnostic test results

- Blood tests, faecal tests, etc.
  - CBC:
    - Hb: 10.8 g/dL (Low)
    - Hct: 33% (Low)
    - MCV: 78 fL (Low)
    - MCH: 25 pg (Low)
    - MCHC: 32 g/dL (Normal)
    - RDW: 15.5% (High)
    - WBC: 7.5 x 10^9/L (Normal)
    - Neutrophils: 4.8 x 10^9/L (64%)
    - Lymphocytes: 2.0 x 10^9/L (27%)
    - Monocytes: 0.5 x 10^9/L (7%)
    - Eosinophils: 0.15 x 10^9/L (2%)
    - Basophils: 0.05 x 10^9/L (<1%)
    - PLT: 350,000 /mm^3 (Normal)
  - Iron Studies:
    - Iron: 30 mcg/dL (Low)
    - Ferritin: 15 ng/mL (Low)
    - TIBC: 450 mcg/dL (High)
    - Transferrin Saturation: 6.7% (Low)
  - Comprehensive Metabolic Panel (CMP):
    - Na: 138 mEq/L (Normal)
    - K: 4.1 mEq/L (Normal)
    - Cl: 102 mEq/L (Normal)
    - CO2: 25 mEq/L (Normal)
    - BUN: 22 mg/dL (Slightly High)

Creatinine: 1.1 mg/dL (Normal for age/muscle mass)

eGFR: >60 mL/min/1.73 m2

Glucose: 125 mg/dL (Elevated, consistent with DM)

Calcium: 9.0 mg/dL (Normal)

Total Protein: 6.2 g/dL (Low end of Normal)

Albumin: 3.3 g/dL (Low)

Total Bilirubin: 0.7 mg/dL (Normal)

Direct Bilirubin: 0.2 mg/dL (Normal)

AST (SGOT): 28 U/L (Normal)

ALT (SGPT): 30 U/L (Normal)

ALP: 95 U/L (Normal)

GGT: 40 U/L (Normal)

Inflammatory Markers:

ESR: 35 mm/h (Elevated)

CRP: 1.5 mg/dL (Slightly Elevated)

Coagulation:

PT/INR: 1.1 (Normal)

PTT: 30 sec (Normal)

Thyroid Function:

TSH: 2.5 mcU/mL (Normal)

fT4: 1.4 ng/dL (Normal)

Tumor Markers:

CEA: 6.0 ng/mL (Elevated, normal <2.5 ng/mL for non-smokers, <5.0 ng/mL for smokers/former)

CA 19-9: 45 U/mL (Elevated, normal <37 U/mL)

Vitamin B12: 450 pg/mL (Normal)

Serum Folate: 8 ng/mL (Normal)

HbA1c: 7.2% (Reflects recent glycemic control)

Fecal Occult Blood Test (FOBT): Positive

Endoscopic test reports

EGDS (Esophagogastroduodenoscopy):

Indication: Unexplained weight loss, early satiety, epigastric discomfort, iron deficiency anemia, positive FOBT.

Esophagus: Normal caliber and mucosa throughout. No signs of esophagitis, varices, or masses. Z-line appeared regular at 38 cm from incisors.

Stomach: Large, ulcerated, infiltrative mass identified along the lesser curvature, extending from the mid-body towards the antrum. The mass measures approximately 5-6 cm in greatest dimension, with raised, irregular borders and a necrotic, friable base. The surrounding gastric folds appear thickened and effaced. The lesion bleeds easily on contact. Multiple biopsies (8 samples) taken from the edge and base of the mass. Remainder of the gastric fundus, cardia, and greater curvature appear grossly unremarkable, though distensibility seems slightly reduced. Pylorus visualized, appears patent but potentially narrowed due to proximity of the antral extension of the mass.

Duodenum: Duodenal bulb and second portion explored. Mucosa appears normal with preserved villous pattern. No ulcers or masses identified.

Conclusion: Large, ulcerated gastric mass highly suspicious for malignancy. Biopsies obtained.

Colonoscopy:

Indication: Age-appropriate screening, positive FOBT, weight loss.

Preparation: Excellent. Boston Bowel Prep Score 9 (3+3+3).

Extent: Examined to the cecum, confirmed by visualization of appendiceal orifice and ileocecal valve. Terminal ileum intubated for 10 cm, appeared normal.

Findings:

Rectum & Sigmoid: Normal mucosa.

Descending Colon: One 5mm sessile polyp found, removed via cold snare polypectomy.

Transverse Colon: Normal mucosa.

Ascending Colon & Cecum: Normal mucosa.

Conclusion: Single diminutive polyp in descending colon removed. No evidence of malignancy or synchronous lesions. Normal terminal ileum.

Histology reports

Gastric Biopsies (Lesser curvature mass):

Microscopic Description: Sections show fragments of gastric mucosa extensively infiltrated by poorly differentiated adenocarcinoma. Malignant glands are irregular, fused, and invade the lamina propria and submucosa (extent limited by biopsy depth). Tumor cells exhibit marked nuclear pleomorphism, hyperchromasia, prominent nucleoli, and frequent mitotic figures. Some signet ring cells are identified focally. Associated desmoplastic stromal reaction and inflammatory infiltrate are present. No Helicobacter pylori organisms identified on special stains.

Diagnosis: Poorly Differentiated Adenocarcinoma of the Stomach, with signet ring features.

Colon Polyp (Descending colon):

Microscopic Description: Sections show fragments of colonic mucosa forming a tubular adenoma. Glandular architecture is maintained but cells show low-grade dysplasia characterized by nuclear stratification, hyperchromasia, and elongation. No high-grade dysplasia or invasive carcinoma identified. Resection margins appear clear.

Diagnosis: Tubular Adenoma with Low-Grade Dysplasia.

Imaging reports

Abdominal Ultrasound (Initial, before endoscopy):

Liver: Normal size and echotexture. No focal lesions identified. Portal vein patent. No intra or extrahepatic biliary dilatation.

Gallbladder: Contracted (patient not fasting adequately). No definite gallstones or wall thickening seen. Common bile duct measures 4 mm (normal).

Pancreas: Partially obscured by overlying bowel gas in the head and tail region. Visualized portions of the body appear unremarkable. Pancreatic duct not dilated.

Spleen: Normal size and echotexture.

Kidneys: Normal size, position, and echotexture bilaterally. No hydronephrosis or calculi.

Aorta/IVC: Normal caliber.

Ascites: None detected.

Conclusion: Limited visualization of the pancreas. No specific abnormalities detected to explain symptoms, though gastric pathology cannot be excluded by ultrasound. Recommend further evaluation as clinically indicated.

Abdominal/Pelvic CT Scan with IV and Oral Contrast (Staging scan after EGD):

Technique: Axial images obtained before and after administration of IV omnipaque contrast and oral positive contrast. Coronal and sagittal reformats provided.

Findings:

Stomach: Marked circumferential wall thickening involving the gastric body and antrum, predominantly along the lesser curvature, consistent with the known malignancy. The thickened wall measures up to 2.5 cm. Evidence of ulceration is noted. The adjacent perigastric fat shows stranding and several enlarged lymph nodes (largest measuring 1.8 cm in short axis) are seen in the gastrohepatic ligament and peripancreatic region.

Liver: Multiple hypodense lesions seen scattered throughout both lobes, consistent with metastatic disease. The largest lesion in segment VII measures 3.5 cm. No biliary dilatation.

Pancreas: Appears normal in size and enhancement. No definite invasion by gastric tumor, but enlarged nodes abut the pancreatic body.

Spleen, Adrenals, Kidneys: Unremarkable.

Bowel: No evidence of obstruction. Colon appears normal aside from post-polypectomy changes.

Lymph Nodes: In addition to perigastric nodes, borderline enlarged retroperitoneal nodes are noted.

Ascites: Trace amount of perihepatic ascites.

Bones: No suspicious osseous lesions identified.

Lungs: Lung bases visualized show no acute abnormalities.

Impression:

1. Large infiltrative gastric mass consistent with known adenocarcinoma.
2. Significant perigastric and regional lymphadenopathy.
3. Multiple liver lesions highly suspicious for metastatic disease.
4. Trace ascites.
5. Findings consistent with Stage IV (metastatic) gastric adenocarcinoma.

Chest CT Scan (Staging, performed with Abdominal/Pelvic CT):

Technique: Axial images obtained without IV contrast through the thorax.

Findings:

Lungs: No definite pulmonary nodules or masses. No consolidation or pleural effusion.

Mediastinum: No pathologically enlarged mediastinal or hilar lymph nodes. Heart size normal.

Bones: Visualized portions of the thoracic skeleton show degenerative changes, no suspicious metastatic lesions.

Impression: No evidence of thoracic metastatic disease.

Other tests, if applicable

PET/CT Scan (Optional, depending on treatment plan consideration, but logical next step for refining staging/metastatic burden):

Technique: Images acquired after IV administration of F-18 FDG. Low-dose CT used for attenuation correction and anatomical localization.

Findings:

Primary Tumor: Intense FDG uptake within the thickened gastric wall corresponding to the known malignancy (SUVmax 14.5).

Lymph Nodes: Intense FDG uptake in multiple perigastric, gastrohepatic ligament, peripancreatic, and retroperitoneal lymph nodes (SUVmax range 8.0 - 12.0).

Liver: Multiple hepatic lesions demonstrate intense FDG uptake, confirming metabolic activity consistent with metastases (SUVmax up to 11.5).

Other: Mild diffuse uptake in the colon consistent with physiological activity. No other suspicious distant FDG-avid foci identified in the chest, abdomen, or pelvis.

Impression: Hypermetabolic gastric malignancy with extensive hypermetabolic regional lymphadenopathy and multiple hypermetabolic liver metastases, consistent with Stage IV disease.

Diagnosis

Correct diagnosis: Metastatic Gastric Adenocarcinoma (Stage IV) with liver and regional lymph node metastases.

Optimal treatment options:

Palliative Intent (given Stage IV disease):

Systemic Chemotherapy: Combination chemotherapy regimen (e.g., FLOT [Fluorouracil, Leucovorin, Oxaliplatin, Docetaxel] if fit, or doublet regimens like FOLFOX [Fluorouracil, Leucovorin, Oxaliplatin] or CAPOX [Capecitabine, Oxaliplatin], or FOLFIRI [Fluorouracil, Leucovorin, Irinotecan] depending on performance status and comorbidities). HER2 testing of tumor tissue should be performed; if positive, Trastuzumab may be added to chemotherapy. PD-L1 testing should also be considered for potential immunotherapy options (e.g., Nivolumab or Pembrolizumab) in first-line or subsequent lines.

Nutritional Support: Consultation with a dietitian. Consideration of nutritional supplements. Management of early satiety (small, frequent meals). Potential need for feeding tube (e.g., G-J tube) if oral intake remains inadequate or gastric outlet obstruction develops.

Symptom Management:

Pain control (analgesics).

Management of anemia (iron supplementation, potential transfusions).

Management of potential gastric outlet obstruction (endoscopic stenting if needed).

Management of nausea/vomiting (antiemetics).

Palliative Radiotherapy: May be considered for symptomatic relief of specific metastatic sites (e.g., painful bone metastases, though none identified here) or for bleeding control from the primary tumor.

End-of-Life Care Planning/Advance Directives: Discussion regarding goals of care.

Follow-up proposal

Follow-up tests and timing:

CBC, CMP: Before each cycle of chemotherapy and as clinically indicated.

Tumor Markers (CEA, CA 19-9): Can be monitored during therapy, though imaging is more reliable for assessing response (usually every 2-3 months).

CT Scans (Chest/Abdomen/Pelvis): Restaging scans typically performed every 8-12 weeks during chemotherapy to assess treatment response.

Follow-up consultations and timing:

Medical Oncology: Primary managing service. Follow-up every 2-4 weeks initially during chemotherapy, then as needed based on treatment plan and patient status.

Gastroenterology: As needed for endoscopic interventions (e.g., stent placement) or management of GI symptoms.

Palliative Care: Consultation early for symptom management, goals of care discussion, and psychosocial support.

Nutrition: Regular follow-up to monitor nutritional status and adjust support.

Primary Care Physician: Ongoing management of comorbidities (Diabetes, Hypertension).

Monitoring for complications:

Disease Progression: Worsening symptoms, new sites of metastasis on imaging.

Treatment Toxicity: Side effects of chemotherapy (nausea, vomiting, diarrhea, neuropathy, myelosuppression).

Gastric Outlet Obstruction: Worsening early satiety, nausea, vomiting undigested food.

Tumor Bleeding: Melena, hematemesis, worsening anemia.

Malnutrition/Cachexia: Ongoing weight loss, muscle wasting.

Thromboembolism: Increased risk in cancer patients.

Clinical Case ID: 11

Medical history

- Age and sex: 42-year-old female
- Reason for seeking medical consultation: Approximately 2-year history of bothersome alternating bowel habits (constipation and diarrhea), associated with abdominal cramping and bloating. Symptoms have worsened over the last 6 months, impacting daily activities.

All symptoms/complaints:

- Alternating Bowel Habits: Describes periods lasting several days to weeks of difficult-to-pass, hard stools (Bristol Stool Form Scale Type 1-2), occurring ≤3 times per week, often with a sensation of incomplete evacuation. These periods alternate with phases lasting several days characterised by loose, watery stools (Bristol Type 6-7), occurring ≥3 times per day, sometimes with urgency. Current pattern is roughly 1 week constipation followed by 3-4 days diarrhea.
- Abdominal Pain: Cramping pain, described as moderate intensity (4-6/10), located diffusely across the lower abdomen but sometimes migrating. Pain is often worse before a bowel movement and typically improves temporarily after defecation (either constipation or diarrhea phase).
- Bloating: Reports significant abdominal bloating and a sensation of distension, particularly worse towards the end of the day and during constipation phases.
- Mucus in Stool: Occasionally notices mucus in the stool, especially during diarrhea phases. No visible blood reported.
- Other: No significant weight loss, fever, night sweats, rectal bleeding, or persistent fatigue beyond what she attributes to poor sleep related to discomfort. Appetite is generally normal. Notes symptoms worsen during periods of increased stress (work deadlines).

- Results of diagnostic tests already done (1 year ago, via GP):
    - Complete Blood Count (CBC): Reported as "within normal limits".
    - Thyroid Stimulating Hormone (TSH): Reported as "normal".

- Comorbidities/previous surgery/etc. (APR):
    - History of migraines (managed with sumatriptan as needed)

- Mild seasonal allergies
- Cholecystectomy 5 years ago for symptomatic gallstones.

- Family history:
    - Mother has "sensitive stomach", unspecified diagnosis.
    - Father has hypertension.
    - No family history of inflammatory bowel disease, celiac disease, or colorectal cancer.

- Pharmacological history (and allergies):
    - Sumatriptan 50mg PRN for migraines (uses 1-2 times per month).
    - Loratadine 10mg PRN for seasonal allergies.
    - Occasionally uses over-the-counter polyethylene glycol for constipation and loperamide for diarrhea (self-medicating).
    - No known drug allergies.

- Risk factors and diet:
    - Smoking status: Never smoked.
    - Alcohol status: Social drinker (1-2 glasses of wine per week).
    - Body weight: 65 kg, Height: 168 cm (BMI 23.0 kg/m² - stable).
    - Diet type: Reports eating a "generally healthy" diet but finds certain foods (e.g., beans, onions, sometimes dairy) seem to worsen bloating and gas. High-stress job in finance. Exercises moderately (walks 3-4 times/week).

- Physical examination:
    - Vitals: BP 124/78 mmHg, HR 72 bpm, RR 16/min, Temp 37.0°C (98.6°F).
    - General: Alert, oriented, appears well-nourished, in no acute distress.
    - Abdomen: Soft, non-distended. Mild diffuse tenderness to palpation in lower quadrants, particularly left lower quadrant, without guarding or rebound tenderness. Bowel sounds are normoactive. No hepatosplenomegaly. Murphy's sign negative. Surgical scar in RUQ well-healed.
    - Rectal Exam: Normal external appearance. Good sphincter tone. Vault empty. No masses palpated. Stool guaiac test negative.

Diagnostic test results

- Blood tests, faecal tests, etc.
  - CBC:
    - Hb 13.8 g/dL
    - MCV 91 fl
    - WBC 6.5 x 10^9/L (Neutrophils 55%, Lymphocytes 35%, Monocytes 6%, Eosinophils 3%, Basophils 1%)
    - PLT 250,000 /mm^3
  - CMP:
    - Na 141 mEq/L
    - K 4.2 mEq/L
    - Cl 102 mEq/L
    - HCO3 25 mEq/L
    - BUN 14 mg/dL
    - Creatinine 0.78 mg/dL (eGFR >90 mL/min/1.73 m^2)
    - Glucose 92 mg/dL
    - Ca 9.5 mg/dL
    - Total Protein 7.2 g/dL
    - Albumin 4.5 g/dL
    - ALT 28 U/L
    - AST 24 U/L
    - ALP 65 U/L
    - Total Bilirubin 0.6 mg/dL
  - Inflammatory Markers:
    - ESR: 12 mm/h
    - CRP: <0.5 mg/dL
  - Thyroid Function:
    - TSH: 2.1 mcU/mL (confirms previous normal)
    - fT4: 1.3 ng/dL
  - Celiac Serology:
    - Tissue Transglutaminase (tTG) IgA: 3 U/mL (<15 U/mL normal)
    - Total IgA: 210 mg/dL (normal range 70-400 mg/dL)

- Stool Tests:
    - Stool Culture (for Salmonella, Shigella, Campylobacter, E. coli O157:H7): Negative
    - Ova and Parasites Exam (x3): Negative
    - Clostridium difficile Toxin Assay: Negative
    - Fecal Calprotectin: 45 mcg/g (<50 mcg/g considered normal)
    - Fecal Elastase: 350 mcg/g (>200 mcg/g normal)
    - Fecal Fat Qualitative: Negative

- Endoscopic test reports
  - EGDS:
    - Indication: Evaluation of upper abdominal symptoms (bloating, though primary complaint is lower GI).
    - Findings:
      - Esophagus: Normal mucosa throughout. No signs of esophagitis, Barrett's esophagus, varices, or masses. Z-line regular at 39 cm from incisors.
      - Stomach: Normal gastric mucosa in fundus, body, and antrum. No erosions, ulcers, polyps, or masses. Small amount of clear bile noted in the fundus (consistent with post-cholecystectomy status). Pylorus is patent and normal.
      - Duodenum: Normal appearance of duodenal bulb and second portion. Villi appear grossly normal.
    - Biopsies: Taken from distal esophagus, gastric antrum, and duodenum (standard protocol).
    - Conclusion: Normal upper endoscopy.

  - Colonoscopy:
    - Indication: Evaluation of alternating bowel habits, abdominal pain, rule out inflammatory bowel disease or malignancy.
    - Preparation Quality: Excellent (Boston Bowel Preparation Scale 9/9).
    - Extent of Exam: Reached cecum. Terminal ileum intubated for 15 cm.
    - Findings:
      - Terminal Ileum: Normal mucosal appearance. No ulcerations or inflammation.

- Colon: Entire colon from cecum to rectum visualized. Normal vascular pattern, haustral folds, and mucosal appearance throughout. No polyps, masses, diverticula, inflammation, ulcerations, or evidence of colitis observed. Anus and rectum appear normal.

- Biopsies: Random biopsies taken from terminal ileum, cecum, ascending colon, transverse colon, descending colon, sigmoid colon, and rectum (to rule out microscopic colitis).

- Conclusion: Normal colonoscopy to the terminal ileum.

- SBCE (Small Bowel Capsule Endoscopy):

- Indication: Considered due to persistent symptoms despite negative endoscopies, but deferred as clinical picture highly suggestive of functional disorder. (If ordered hypothetically):

- Procedure: Patient swallowed capsule without difficulty. Complete transit to cecum confirmed. Transit time: 4 hours 15 minutes.

- Findings: Normal mucosal appearance throughout the duodenum, jejunum, and ileum. No evidence of ulcerations, erosions, strictures, masses, vascular malformations, or active bleeding.

- Conclusion: Normal small bowel capsule endoscopy.

- Imaging Reports

- Abdominal Ultrasound (US):

- Indication: General evaluation of abdominal pain, rule out residual biliary issues post-cholecystectomy, evaluate solid organs.

- Findings:

- Liver: Normal size, echotexture. No focal lesions. No intrahepatic biliary dilatation.

- Gallbladder: Surgically absent. Common bile duct measures 4 mm (normal).

- Pancreas: Head, body, and tail appear normal in size and echotexture. Pancreatic duct not dilated.

- Spleen: Normal size and echotexture.

- Kidneys: Normal size, position, and echogenicity. No hydronephrosis or calculi.

- Aorta/IVC: Normal caliber.

- Other: No free fluid detected. Bowel loops appear unremarkable with normal peristalsis observed, though assessment is limited by bowel gas.

- Conclusion: Normal abdominal ultrasound. Status post cholecystectomy.

- Abdominal/Pelvic CT Scan (with contrast):

- Indication: Performed primarily to exclude structural abnormalities given chronicity of symptoms and patient anxiety, although low pre-test probability.

- Findings:

- Liver, Spleen, Pancreas, Adrenal Glands, Kidneys: All appear normal in size, morphology, and enhancement. No focal lesions.

- Biliary Tree: No intra- or extrahepatic biliary dilatation. CBD normal caliber. Gallbladder absent.

- Bowel: Stomach, small bowel loops, and colon appear normal in caliber and wall thickness. No evidence of bowel obstruction, significant wall thickening suggestive of inflammation, diverticulitis, or masses. Appendix not visualized (assumed normal if present, or status post removal if applicable - check APR, none noted). Minimal stool burden in the colon.

- Vasculature: Aorta, IVC, mesenteric vessels appear patent without significant stenosis or thrombosis.

- Lymph Nodes: No pathological lymphadenopathy identified.

- Pelvic Organs: Uterus and ovaries appear normal for age. No adnexal masses. Bladder unremarkable.

- Other: No ascites or significant free air. Osseous structures unremarkable.

- Conclusion: Normal abdominal and pelvic CT scan.

- Abdominal MRI/Entero-MRI:

- Indication: Generally not indicated given negative prior workup, but if performed:

- Findings: Would confirm normal appearance of solid organs, biliary tree, and vasculature. Small bowel and large bowel loops would show normal caliber, wall thickness (<3mm), and enhancement pattern. No evidence of strictures, fistulas, inflammatory changes (e.g., edema, fat stranding), or masses.

- Conclusion: Normal abdominal MRI/Entero-MRI (hypothetical).

- Histology reports

- Esophageal Biopsy: Squamous mucosa with no significant inflammation, intestinal metaplasia, or dysplasia.

- Gastric Biopsy (Antrum): Mild chronic inactive gastritis. No Helicobacter pylori organisms identified (negative immunohistochemical stain). No intestinal metaplasia or atrophy.
  - Duodenal Biopsy: Normal villous architecture (villous:crypt ratio approx. 4:1). Intraepithelial lymphocyte count within normal limits (<25 IELs per 100 enterocytes). No evidence of celiac disease, giardiasis, or other specific duodenitis.
  - Colonic Biopsies (Multiple Sites including Terminal Ileum):
    - Terminal Ileum: Normal villous architecture. No significant inflammation or granulomas.
    - Colon (Cecum, Ascending, Transverse, Descending, Sigmoid, Rectum): Normal colonic mucosa at all sites. Crypt architecture is preserved. No increase in lamina propria cellularity, no cryptitis, crypt abscesses, granulomas, or evidence of microscopic colitis (collagenous or lymphocytic). Normal number of intraepithelial lymphocytes.

- Other tests, if applicable
  - Hydrogen Breath Test (Lactulose or Glucose):
    - Indication: To assess for Small Intestinal Bacterial Overgrowth (SIBO), which can cause bloating and altered bowel habits.
    - Result (Hypothetical - often considered): Negative. No early rise in hydrogen or methane after lactulose/glucose administration meeting criteria for SIBO.
  - Hydrogen Breath Test (Lactose/Fructose):
    - Indication: To assess for malabsorption potentially contributing to symptoms.
    - Result (Hypothetical - if specific food triggers suspected): Could be positive for either lactose or fructose malabsorption, suggesting these might be dietary triggers, but doesn't exclude underlying IBS. For this case, assume Negative for both.

Diagnosis

- Correct diagnosis: Irritable Bowel Syndrome with Mixed Bowel Habits (IBS-M) based on Rome IV criteria:
  - Recurrent abdominal pain, on average, at least 1 day/week in the last 3 months.
  - Associated with 2 or more of the following:
    - Related to defecation (pain often improves post-BM).
    - Associated with a change in frequency of stool (alternating between <3/week and >3/day).

- Associated with a change in form (appearance) of stool (alternating between Bristol Type 1-2 and 6-7).

- Criteria fulfilled for the last 3 months with symptom onset at least 6 months prior to diagnosis.

- Absence of red flag symptoms (significant weight loss, rectal bleeding, nocturnal symptoms, family history of IBD/CRC) and extensive negative workup excluding organic disease (inflammatory, infectious, celiac, malignancy, significant structural abnormalities).

Optimal treatment options:

1. Patient Education and Reassurance: Explain the nature of IBS as a chronic functional disorder of gut-brain interaction, not a structural or progressive disease. Reassure based on negative workup.

2. Lifestyle Modifications:

- Stress Management: Techniques like cognitive behavioral therapy (CBT), relaxation exercises, mindfulness, yoga. Identify and manage stressors.

- Regular Exercise: Moderate physical activity can improve bowel function and mood.

- Sleep Hygiene: Ensure adequate sleep.

3. Dietary Modifications:

- Initial Approach: Keep a food and symptom diary. Avoid common triggers identified (e.g., gas-producing foods like beans, onions, cruciferous vegetables, excessive caffeine, alcohol, fatty foods).

- Low FODMAP Diet: Consider a trial of a dietitian-supervised Low FODMAP (Fermentable Oligo-, Di-, Mono-saccharides And Polyols) diet for 4-6 weeks, followed by systematic reintroduction to identify specific triggers.

- Fiber Management: Titrate soluble fiber (e.g., psyllium) carefully; start low and go slow. Insoluble fiber (e.g., bran) may worsen pain/bloating in some IBS patients. Adjust based on constipation/diarrhea phase.

4. Pharmacological Therapy (Symptom-Directed):

- For Pain/Cramping: Antispasmodics (e.g., Dicyclomine 10-20mg QID PRN, Hyoscyamine 0.125mg SL/PO PRN or TID/QID). Peppermint oil capsules (enteric-coated).

- For Constipation Phases: Osmotic laxatives (e.g., Polyethylene glycol 3350, 17g daily PRN), Lubiprostone (Amitiza) 8mcg BID, Linaclotide (Linzess) 72-145mcg daily, Plecanatide (Trulance) 3mg daily.

- For Diarrhea Phases: Loperamide (Imodium) 2-4mg PRN (up to 16mg/day), used cautiously. Eluxadoline (Viberzi) 75-100mg BID (use with caution, especially post-cholecystectomy due to

pancreatitis risk - requires careful discussion). Rifaximin (Xifaxan) 550mg TID for 14 days (for bloating/diarrhea, may need repeat courses). Bile acid sequestrants (e.g., Cholestyramine) if post-cholecystectomy diarrhea suspected, though less likely given alternating pattern.

- For Global Symptoms/Pain/Mood: Low-dose Tricyclic Antidepressants (TCAs) (e.g., Amitriptyline 10-25mg QHS, Nortriptyline 10-25mg QHS) or SSRIs (e.g., Citalopram, Fluoxetine) can modulate visceral hypersensitivity and improve mood.

Follow-up proposal

- Follow-up tests and timing:
    - Generally, no routine repeat testing needed unless red flag symptoms develop or clinical picture changes significantly.
    - Monitor CBC/CMP periodically (e.g., annually) if on certain long-term medications or if symptoms warrant.
- Follow-up consultations and timing:
    - Gastroenterology: Follow up in 4-8 weeks to assess response to initial interventions (lifestyle, diet, +/- initial medications). Subsequent follow-up every 3-6 months depending on symptom control and patient needs.
    - Registered Dietitian: Essential for guidance on Low FODMAP diet implementation and reintroduction phase, and long-term dietary management.
    - Mental Health Professional: Consider referral for CBT or other stress management therapies if stress is a significant trigger or if mood is significantly affected.
- Monitoring for complications:
    - IBS itself does not lead to serious physical complications like cancer or IBD. Monitoring focuses on symptom control, quality of life, and impact on daily functioning.
    - Monitor for development of red flag symptoms that would prompt re-evaluation.
- Educate patient on appropriate use of PRN medications to avoid side effects (e.g., rebound constipation with excessive loperamide).

Clinical Case ID: 17

Medical history

- Age and sex: 37-year-old female
- Reason for seeking medical consultation: Persistent dyspepsia over the last 4 months, recently confirmed positive for Helicobacter pylori via stool antigen test ordered by her primary care physician.

All symptoms/complaints:

- Dyspepsia: Described as a burning or gnawing sensation localized to the epigastrium. Occurs most days, typically starting 1-2 hours after meals. Worse after eating spicy foods, fatty meals, or drinking coffee. Sometimes awakens her in the early morning hours (around 2-3 AM). Partially relieved by over-the-counter antacids (calcium carbonate), but relief is temporary (lasts < 1 hour).
- Bloating: Frequent sensation of fullness and pressure in the upper abdomen, especially after meals.
- Early Satiety: Feeling full quickly after starting to eat, unable to finish normal-sized meals occasionally.
- Nausea: Mild, intermittent nausea, particularly associated with the epigastric burning. No vomiting.
- No heartburn/regurgitation suggestive of GERD.
- No dysphagia or odynophagia.
- No change in bowel habits (no diarrhea, constipation, melena, or hematochezia reported).
- No significant weight loss reported; appetite is slightly reduced due to early satiety and fear of provoking pain.
- No fever, chills, or night sweats.

Results of diagnostic tests already done (1 week ago by PCP):

- Helicobacter pylori Stool Antigen Test: Positive

Comorbidities/previous surgery/etc. (APR):

- Generalized Anxiety Disorder (GAD), diagnosed 5 years ago, managed with medication.
- History of migraines, infrequent (1-2 times per year).

- Cholecystectomy (laparoscopic) 10 years ago for symptomatic gallstones.
- Tonsillectomy as a child.

Family history:
- Mother: Diagnosed with a peptic ulcer in her 40s (details unknown, presumed H. pylori related).
- Father: Hypertension, Type 2 Diabetes Mellitus.
- No known family history of gastric cancer or other GI malignancies.

Pharmacological history (and allergies):
- Sertraline 100 mg daily (for GAD)
- Sumatriptan 50 mg as needed (for migraines)
- Calcium Carbonate (Tums) as needed for dyspepsia (uses 4-5 tablets/day recently)
- Allergy: Penicillin (causes hives)

Risk factors and diet:
- Smoking status: Never smoked.
- Alcohol status: Social drinker, 2-3 glasses of wine per week.
- NSAID use: Avoids NSAIDs due to awareness of potential stomach upset; uses acetaminophen for occasional headaches.
- Body weight: 68 kg, Height: 165 cm (BMI 25.0 kg/m^2) - stable weight.
- Diet type: Describes diet as generally balanced but admits to frequent coffee consumption (3-4 cups/day) and enjoys spicy foods. Irregular meal timings due to busy work schedule.

Physical examination:
- Vitals: BP 124/78 mmHg, HR 76 bpm, RR 16/min, Temp 36.8°C (98.2°F), SpO_2 99% on room air.
- General: Alert, oriented, in no acute distress. Appears well-nourished.
- Abdomen: Soft, non-distended. Mild tenderness to deep palpation in the epigastric region. No guarding or rebound tenderness. Bowel sounds normal. No hepatosplenomegaly. Murphy's sign negative. Scar from previous laparoscopic cholecystectomy visible, well-healed.
- Cardiovascular: Regular rate and rhythm, no murmurs.
- Respiratory: Clear to auscultation bilaterally.
- Skin: No jaundice, pallor, or significant rashes.

Diagnostic test results

- Blood tests, faecal tests, etc.
  - Hb 13.5 g/dL, MCV 90 fl
  - WBC 7.2 x 10^9/L, neutrophils 4.5 x 10^9/L (62.5%), lymphocytes 2.0 x 10^9/L (27.8%), monocytes 0.5 x 10^9/L (6.9%), eosinophils 0.15 x 10^9/L (2.1%), basophils 0.05 x 10^9/L (0.7%)
  - PLT 250,000 /mm^3
  - Iron: 80 mcg/dL
  - Ferritin: 75 mcg/L
  - Transferrin Saturation: 30%
  - Vitamin B12 650 pg/mL, serum folates 12 ng/mL
  - ESR: 10 mm/h
  - CRP: <0.5 mg/dL
  - ALT 20 U/L, AST 18 U/L, GGT 25 U/L, ALP 65 U/L
  - Total bilirubin 0.7 mg/dL, direct bilirubin 0.2 mg/dL
  - Creatinine 0.75 mg/dL, eGFR >90 mL/min/1.73 m2
  - Amylase 70 U/L, lipase 50 U/L
  - Total cholesterol 180 mg/dL, LDL cholesterol 110 mg/dL, HDL cholesterol 55 mg/dL, serum triglycerides 75 mg/dL
  - Serum glucose 92 mg/dL (fasting)
  - Na 141 mEq/L, K 4.2 mEq/L, Ca 9.5 mg/dL, Mg 2.0 mg/dL
  - Total serum protein 7.5 g/dL, albumin 4.5 g/dL
  - Serology for Celiac Disease (ordered due to dyspepsia, though low suspicion):
    - IgA anti-tissue transglutaminase (tTG): <2 U/mL (Negative)
    - Total IgA 220 mg/dL (Normal)
  - Fecal Occult Blood Test (FOBT): Negative x 3
  - Urea Breath Test (UBT): Not performed yet; planned post-treatment for confirmation of eradication.
  - Fecal Calprotectin: 30 mcg/g (Normal)

- Endoscopic test reports
  - EGDS (Esophagogastroduodenoscopy):

- Indication: Dyspepsia, H. pylori positive status.

- Premedication: Midazolam 3mg IV, Fentanyl 50mcg IV. Topical pharyngeal anesthesia.

- Esophagus: Normal caliber and appearance throughout. Squamocolumnar junction (Z-line) located at 38 cm from incisors, appears regular. No signs of reflux esophagitis, Barrett's esophagus, erosions, ulcers, varices, or masses.

- Stomach: Moderate diffuse erythema, edema, and scattered petechiae noted throughout the gastric antrum and body. Mucosa appears mildly friable. Rugal folds appear slightly thickened. No discrete ulcers, polyps, or masses identified. Pylorus is patent and appears normal. Biopsies taken from antrum (2 samples) and corpus (2 samples) for histology and H. pylori testing.

- Duodenum: Duodenal bulb and second portion of the duodenum visually inspected. Normal appearing mucosa with preserved villous pattern. No ulcerations, erosions, inflammation, or masses. Biopsy taken from duodenal bulb (1 sample).

- Conclusion: Findings consistent with moderate pangastritis. Biopsies obtained.

- Imaging test reports

- Abdominal Ultrasound (performed to rule out residual biliary issues/other causes of upper abdominal pain):

- Liver: Normal size, echogenicity, and contour. No focal lesions identified. Portal vein and hepatic veins are patent with normal flow direction. Intrahepatic bile ducts are not dilated.

- Gallbladder: Surgically absent. Common bile duct measures 4 mm in diameter (normal).

- Pancreas: Head, body, and tail visualized adequately and appear unremarkable. No masses, cysts, or ductal dilatation.

- Spleen: Normal size and echotexture.

- Kidneys: Normal size, position, and echotexture. No hydronephrosis or calculi.

- Aorta/IVC: Normal caliber.

- Peritoneal cavity: No free fluid detected.

- Conclusion: Surgically absent gallbladder. Otherwise, normal ultrasound examination of the upper abdomen.

- Abdominal CT scan (Hypothetical - might be ordered if diagnosis remains unclear or complications suspected, but likely not needed initially):

- Technique: Axial images obtained before and after administration of IV and oral contrast.

- Findings: Liver, spleen, pancreas, adrenal glands, and kidneys demonstrate normal size, density, and enhancement characteristics. No focal lesions or masses identified. Gallbladder is surgically absent. Bile ducts are not dilated. Stomach walls appear mildly and diffusely thickened, consistent with gastritis, but no discrete mass or ulceration evident. Small bowel and colon are unremarkable. No free air or fluid collections. No abdominal or pelvic lymphadenopathy. Visualized portions of the lungs are clear. Osseous structures are unremarkable.

- Conclusion: Mild diffuse gastric wall thickening likely representing gastritis. Surgically absent gallbladder. No other acute intra-abdominal pathology identified.

- Abdominal MRI (Hypothetical - very unlikely for this presentation):

  - Technique: Multi-sequence MR imaging of the abdomen performed with and without IV gadolinium contrast.

  - Findings: Solid organs (liver, pancreas, spleen, kidneys, adrenals) demonstrate normal signal characteristics and morphology. No focal lesions detected. Biliary tree is unremarkable, gallbladder is absent. Stomach shows mild diffuse wall thickening and enhancement, consistent with gastritis. Small and large bowel loops appear normal. No mesenteric abnormalities or lymphadenopathy. Vascular structures are patent. No free fluid.

  - Conclusion: Findings suggestive of gastritis. Surgically absent gallbladder. No evidence of malignancy or other significant intra-abdominal pathology.

- Other tests, if applicable
  - NONE

- Histology reports
  - Gastric Antrum Biopsies (2 samples):

    - Microscopic Description: Sections show gastric antral mucosa with surface foveolar epithelium and underlying glands. There is moderate chronic inflammation within the lamina propria, characterized by lymphocytes and plasma cells. Additionally, there is moderate acute inflammation with neutrophils infiltrating the lamina propria and glandular epithelium (cryptitis and occasional crypt abscesses). Spiral-shaped organisms consistent with Helicobacter pylori are identified on H&E stain and confirmed with Giemsa special stain, present on the surface epithelium and within gastric pits. No intestinal metaplasia, atrophy, or dysplasia is seen.

- Diagnosis: Chronic active gastritis, moderate, with Helicobacter pylori organisms identified.

- Gastric Corpus Biopsies (2 samples):

- Microscopic Description: Sections show gastric corpus (oxyntic) mucosa. Similar inflammatory changes as seen in the antrum are present, consisting of moderate chronic inflammation and moderate activity (neutrophils). Helicobacter pylori organisms are identified with Giemsa stain. Parietal and chief cells appear unremarkable. No atrophy, intestinal metaplasia, or dysplasia is identified.

- Diagnosis: Chronic active gastritis, moderate, with Helicobacter pylori organisms identified.

- Duodenal Bulb Biopsy (1 sample):

- Microscopic Description: Sections show duodenal mucosa with normal villous architecture (villous to crypt ratio approximately 3-4:1). The surface epithelium and brush border are intact. Intraepithelial lymphocyte count is within normal limits (<25 per 100 enterocytes). Brunner's glands are present in the submucosa and appear normal. Lamina propria shows a mild, non-specific chronic inflammatory infiltrate, considered within normal limits. No evidence of active duodenitis, ulceration, celiac disease, or giardiasis.

- Diagnosis: Duodenal bulb mucosa, normal histology.

Diagnosis

- Correct diagnosis: Helicobacter pylori-associated chronic active pangastritis.

Optimal treatment options:

1. H. pylori Eradication Therapy (Penicillin Allergy considerations):

Bismuth Quadruple Therapy (preferred first-line due to Penicillin allergy):

Proton Pump Inhibitor (e.g., Omeprazole 20 mg or Esomeprazole 40 mg) BID

Bismuth subsalicylate 525 mg QID

Metronidazole 500 mg TID or QID

Tetracycline 500 mg QID

Duration: 14 days

Alternative (if Bismuth Quad not tolerated/available, consider local resistance patterns):

Clarithromycin-based Triple Therapy (use Metronidazole instead of Amoxicillin):

PPI (standard dose) BID

Clarithromycin 500 mg BID

Metronidazole 500 mg BID

Duration: 14 days (Note: Higher failure rate if clarithromycin resistance is prevalent).

2. Symptomatic Management:

Continue PPI for 4-8 weeks post-eradication therapy, especially if symptoms persist initially.

Lifestyle modifications: Avoid trigger foods (spicy, fatty), reduce coffee intake, regular meal timings, smoking cessation (if applicable - N/A here), limit alcohol.

Follow-up proposal

- Follow-up tests and timing:

  - Confirmation of Eradication: Urea Breath Test (UBT) or H. pylori Stool Antigen test should be performed at least 4 weeks after completion of antibiotic therapy and at least 2 weeks after stopping PPI therapy to avoid false negatives.

  - Repeat EGD: Generally not required if symptoms resolve and initial EGD showed only gastritis without ulcer, atrophy, metaplasia or dysplasia. Consider if symptoms persist post-successful eradication or if high-risk features were present.

- Follow-up consultations and timing:

  - Gastroenterology: Follow-up visit 6-8 weeks after initiating treatment to review symptom response and arrange/review eradication test results. Further follow-up as needed based on symptom resolution and test results.

  - Primary Care Physician: Routine follow-up for GAD management and general health.

- Monitoring for complications:

  - Educate patient about potential long-term risks if H. pylori is not eradicated (peptic ulcer disease, MALT lymphoma, gastric cancer - although risk stratification needed).

  - Reinforce need for adherence to full course of antibiotics.

- Discuss potential side effects of eradication therapy (e.g., nausea, metallic taste with metronidazole, stool darkening with bismuth).
